# Mpox virus replicates in lung organoids without significantly affecting their cellular function

**DOI:** 10.1016/j.bbrep.2025.102326

**Published:** 2025-11-07

**Authors:** Yoshitaka Nakata, Keiya Uriu, Rina Hashimoto, Takuya Yamamoto, The Genotype to Phenotype Japan (G2P-Japan) Consortium, Akatsuki Saito, Kei Sato, Kazuo Takayama

**Affiliations:** a Department of Synthetic Human Body System, Medical Research Laboratory, Institute of Science Tokyo, Tokyo, 113-8510, Japan; b Division of Systems Virology, Department of Microbiology and Immunology, The Institute of Medical Science, The University of Tokyo 108-8639, Tokyo, Japan; c Center for iPS Cell Research and Application (CiRA), Kyoto University, Kyoto, 606-8507, Japan; d Medical-risk Avoidance Based on iPS Cells Team, RIKEN Center for Advanced Intelligence Project (AIP), Kyoto, 606-8507, Japan; e Institute for the Advanced Study of Human Biology (WPI-ASHBi), Kyoto University, Kyoto, 606-8303, Japan; f Department of Veterinary Science, Faculty of Agriculture, University of Miyazaki, Miyazaki, 889-2192, Japan; g Graduate School of Medicine and Veterinary Medicine, University of Miyazaki, Miyazaki, 889-1692, Japan; h Center for Animal Disease Control, University of Miyazaki, Miyazaki, 889-2192, Japan; i Graduate School of Medicine, The University of Tokyo, Tokyo, 113-8654, Japan; j Graduate School of Frontier Sciences, The University of Tokyo, Kashiwa, 277-0882, Japan; k International Research Center for Infectious Diseases, The Institute of Medical Science, The University of Tokyo, Tokyo, 108-8639, Japan; l International Vaccine Design Center, The Institute of Medical Science, The University of Tokyo, Tokyo, 108-8639, Japan; m Collaboration Unit for Infection, Joint Research Center for Human Retrovirus Infection, Kumamoto University, Kumamoto, 860-0811, Japan; n MRC-University of Glasgow Centre for Virus Research, Glasgow, G61 1AF, UK; o Faculty of Medicine, Chulalongkorn University, Bangkok, 10330, Thailand

**Keywords:** Lung organoids, Mpox virus, iPS cells, Mpox

## Abstract

Patients with mpox may present with a skin rash and mild respiratory symptoms, including sore throat and cough. The genome of the mpox virus (MPXV) has been detected in throat swab specimens from some mpox patients, indicating potential involvement of the respiratory tract. In this study, we used lung organoids to investigate the effects of MPXV infection on the respiratory system by evaluating the viral replication and the infection-mediated host response. MPXV infection resulted in the accumulation of high levels of viral genomes within the cells. H&E staining showed almost no histological differences between MPXV-infected lung organoids and uninfected lung organoids. In addition, RNA-seq analysis revealed that MPXV infection did not significantly alter the gene expression levels of various lung markers. MPXV infection did not change the production of proinflammatory cytokines, including interleukin-1 beta (IL-1β), interleukin-6 (IL-6), tumor necrosis factor-alpha (TNF-α), and interferon-beta (IFN-β). These findings suggest that MPXV can replicate in lung organoids without significantly affecting their cellular function.

## Highlights

• Lung organoids can be used to study MPXV effects on the respiratory tissue.
• Gene expression of lung markers remained unchanged after MPXV infection.
• MPXV infection did not change proinflammatory cytokine production.

## Graphical abstract

**Figure:**
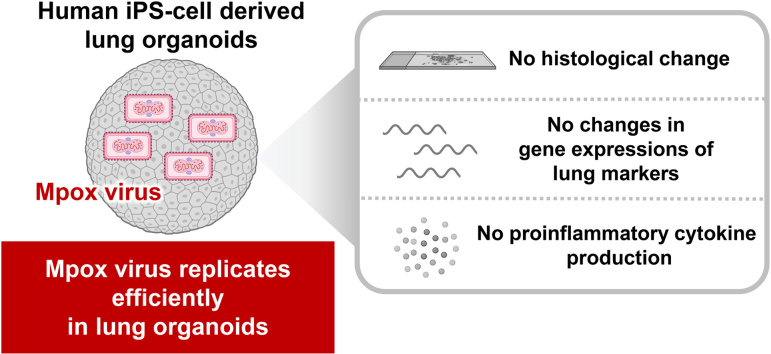

## 1 Introduction

Mpox is a contagious disease caused by the mpox virus (MPXV). MPXV strains are phylogenetically categorized into two primary clades, clade I and II, which are further subdivided into four subclades: clades Ia, Ib, IIa, and IIb. MPXV clades Ia and IIa have been endemic to regions of Central and West Africa [1,2]. In 2022, mpox outbreaks caused by MPXV clade IIb emerged in multiple non-African regions, including the USA, Europe, and Asia. In response, the World Health Organization (WHO) declared a Public Health Emergency of International Concern (PHEIC) in July 2022 [3]. By the time the WHO declared the end of the PHEIC in May 2023, approximately 90,000 mpox cases had been reported globally. The mortality rate and epidemiological characteristics of MPXV differ among its clades, with clade I exhibiting a higher mortality rate compared to clade II [2]. Comparative studies of various MPXV clades are essential to enhance our understanding of the viral characteristics driving outbreaks.

The primary symptoms of mpox include a generalized rash, fever, fatigue, and lymphadenopathy [4]. A meta-analysis reported that respiratory symptoms are observed in approximately 50% of mpox patients [5]. Additionally, the MPXV genome has frequently been detected in throat swab specimens from mpox patients [[6], [7], [8], [9]], suggesting that MPXV may replicate within respiratory tissues. Thus, it is crucial to elucidate the characteristics of MPXV in the lungs and the corresponding host response.

A range of cell and animal models have been employed to characterize MPXV [10]. To analyze host responses in humans, it is crucial to utilize *in vitro* models that accurately replicate human pathophysiology. Since the MPXV outbreak in 2022, characterization of MPXV has been performed using human induced pluripotent stem (iPS) cell and organoid technologies. For instance, human iPS cell-derived colon organoids [11], skin organoids [12], astrocytes, and neural progenitor cells [13] have been utilized. These studies demonstrated that human iPS cell-derived organ models are effective for studying MPXV infection. We have previously developed respiratory models using human iPS cells [14,15] and hypothesize that these models could be instrumental in elucidating the effects of MPXV infection on respiratory tissues. The human iPS cell-derived lung organoids we recently developed contain not only epithelial cells but also macrophages, fibroblasts, and vascular endothelial cells, enabling a more accurate evaluation of host responses to viral infection [15].

In this study, we utilized three MPXV strains, clades Ia, IIa, and IIb, to investigate the virological characteristics of each clade. Additionally, we compared the host response and cellular damage in respiratory tissues caused by MPXV infection among the clades using human iPS cell-derived lung organoids.

## 2 Materials and methods

### 2.1 Human iPS cell culture

The human iPS cell line, 1383D6 [16] (provided by Dr. Masato Nakagawa, Kyoto University), was maintained according to our previous report [11].

### 2.2 Lung organoid differentiation

Lung organoids were differentiated from human iPS cells according to our previous report [14].

### 2.3 MPXV preparation and titration

Mpox virus (MPXV) infection experiments were performed in a biosafety level 3 (BSL3) facility. Three MPXV strains, clade Ia (Zr-599, Genbank accession no. NC_003310.1), clade IIa (Liberia, Genbank accession no. DQ011156.1), and clade IIb (TKY220091, Genbank accession no. LC722946.1), were propagated using VeroE6 cells. Zr-599 and Liberia strains were kindly gifted by the National Institute of Infectious Diseases, Japan. TKY220091 was kindly gifted by the Tokyo Metropolitan Institute of Public Health, Japan. Working MPXV stock was prepared according to our previous report [11]. In addition, virus infectious titers were determined by plaque assay according to our previous report [11]. MPXV infection experiments were performed according to strict regulations in a biosafety level 3 facility at the University of Tokyo.

### 2.4 Quantification and statistical analysis

Statistical significance was evaluated using one- or two-way analysis of variance (ANOVA) followed by Tukey's post hoc tests using GraphPad Prism 9. Details are described in the figure legends.

### 2.5 Data statement

RNA-seq data were submitted under Gene Expression Omnibus (GEO) accession number GSE287262. Other data will be shared upon reasonable request. Requests should be directed to takayama.kazuo@tmd.ac.jp.

## 3 Results

### 3.1 MPXV replicates efficiently in lung organoids

**Fig. 1:**
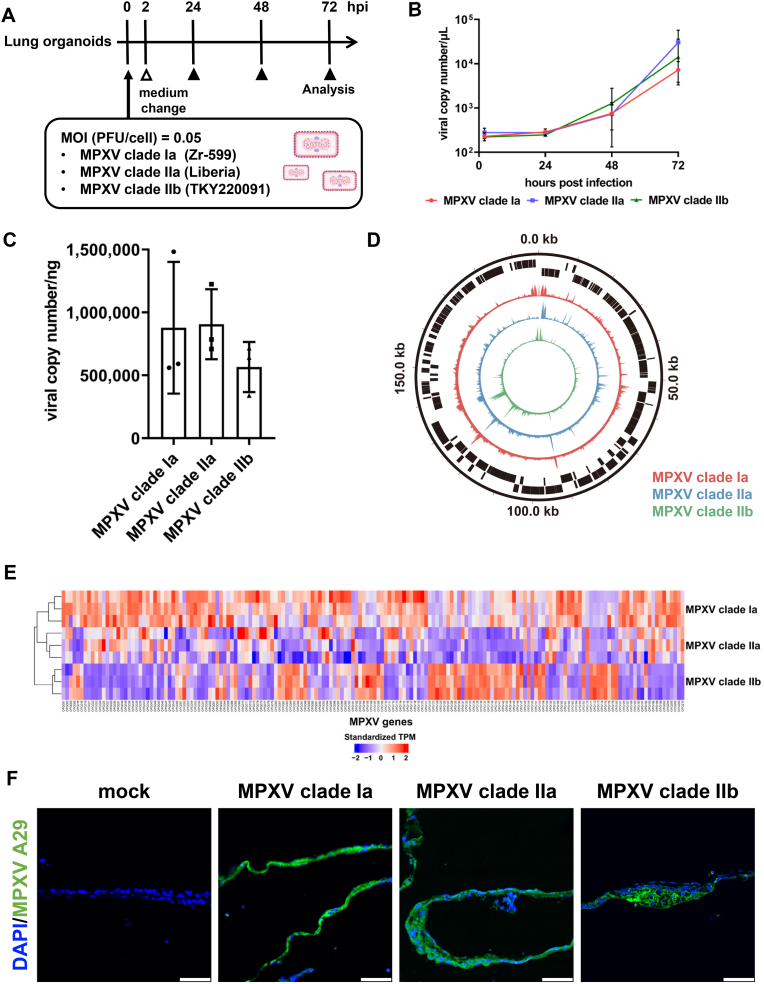
MPXV can propagate in lung organoids MPXV infection experiments in human iPS cell-derived lung organoids. (**A**) The experimental procedure of mpox virus (MPXV) infection experiments. Human iPS cell-derived lung organoids were infected with MPXV (0.05 plaque forming unit (PFU)/cell) and cultured for 72 hpi. Three MPXV strains (clade Ia (Zr-599), clade IIa (Liberia), and clade IIb (TKY220091)) were used in this experiment. (**B**) Viral DNA copy numbers in the cell culture supernatant were measured by qPCR. Two-way analysis of variance (ANOVA) followed by Tukey's post hoc tests. Data are shown as means ± SD (*n* = 6). (**C**) Viral DNA copy numbers in the extracted DNA collected from lung organoids were measured by qPCR. One-way ANOVA followed by the Tukey's post hoc test. Data are shown as means ± SD (*n* = 3). (**D**) RNA-sequencing was performed for uninfected, clade Ia-, clade IIa-, and clade IIb-infected lung organoids. Circos plot showing the distribution of reads along the MPXV clade Ia genome. The inner red, blue, and green circles indicate the reads obtained from MPXV clade Ia-, clade IIa-, and clade IIb-infected lung organoids, respectively. The outer black blocks show the coding regions of MPXV clade Ia. The circos plot was created using pyCirclize ver 1.7.1. (**E**) Heatmap showing the expression profile of MPXV genes. Standardized transcripts per kilobase million (TPM) values of each MPXV gene were visualized and clustered using the “ComplexHeatmap” package [17]. **(F)** Immunofluorescence analysis of MPXV A29 protein (green) in uninfected and MPXV-infected lung organoids. Nuclei were counterstained with DAPI (blue). Scale bars 50 μm.

In this study, we used human iPS cell-derived lung organoids to investigate the effects of MPXV infection on human respiratory tissue. Human iPS cell-derived lung organoids were infected with three MPXV strains, clade Ia (Zr-599), clade IIa (Liberia), and clade IIb (strain TKY220091), and cultured for 72 h (Fig. 1A). MPXV genome levels increased over time in the cell culture supernatants of infected lung organoids (Fig. 1B). The viral copy numbers in the cell culture supernatants at 72 hpi were 7.3 × 10^3^, 3.0 × 10^4^, and 1.4 × 10^4^ copies/μL for MPXV clade Ia, clade IIa, and clade IIb-infected lung organoids, respectively. The number of viral genome copies in the culture supernatants of MPXV-infected lung organoids did not differ significantly between MPXV clades (Table S1). Consistent with this, the number of intracellular viral genome copies in MPXV-infected lung organoids did not differ significantly between MPXV clades (Fig. 1C–Table S1). These results suggest that all MPXV clades replicate efficiently in lung organoids. In addition, to analyze the MPXV gene expression profile in lung organoids, we conducted RNA-seq analysis on MPXV-infected lung organoids. Circos plots displaying the distribution of reads along the MPXV genome revealed high expression of MPXV genes from all clades in lung organoids (Fig. 1D). Clustering analysis showed that the MPXV gene expression profile in lung organoids infected with MPXV clade IIb was distinct from those infected with MPXV clade Ia or IIa (Fig. 1E). Immunofluorescence analysis revealed that MPXV A29-positive cells were observed in the MPXV-infected lung organoids (Fig. 1F). These results demonstrate that MPXV efficiently replicates in lung organoids, with MPXV genes being highly expressed in the infected lung organoids.

### 3.2 Structure and gene expression profile of MPXV-infected lung organoids

**Fig. 2:**
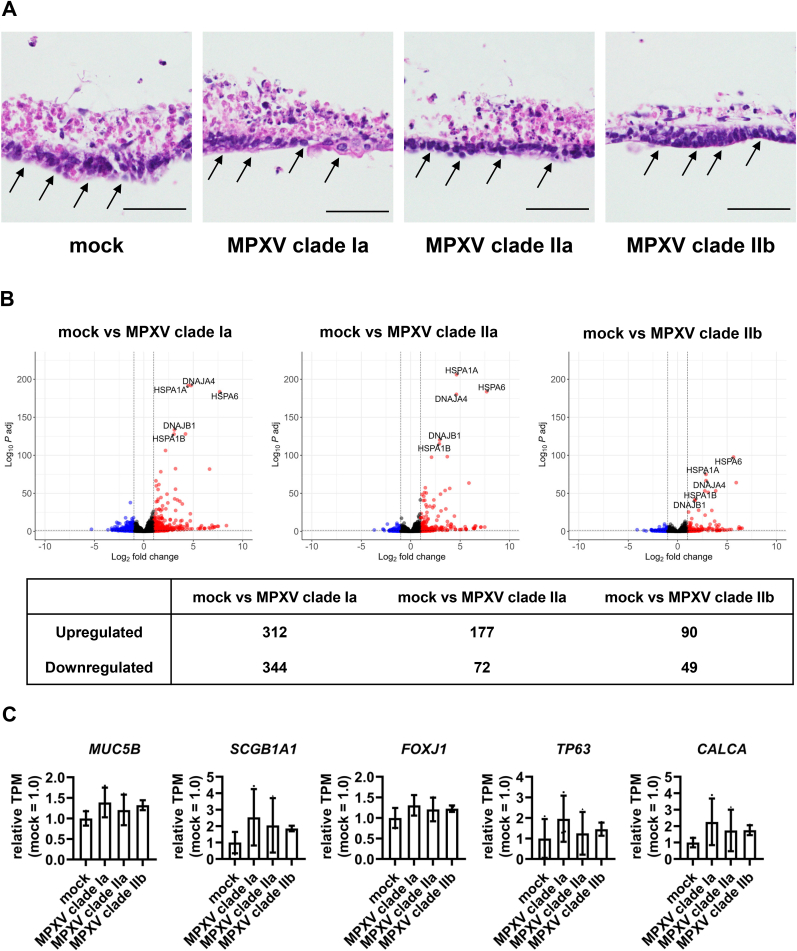
MPXV infection has little effect on morphology and gene expression in lung organoids Human iPS cell-derived lung organoids were infected with MPXV (0.05 PFU/cell) and cultured for 72 hpi. At 72 hpi, characterization of the MPXV-infected lung organoids was performed. (**A**) Hematoxylin and eosin (H&E)-stained images of uninfected and MPXV-infected lung organoids. Brack arrows indicate the epithelial cells. Scale bars 50 μm. (**B**) RNA-seq analysis was performed in uninfected and MPXV-infected lung organoids. Volcano plots showing differentially expressed genes (DEGs) between uninfected and MPXV-infected lung organoids (Log_2_ fold change >1.0, adjusted p-value (*P* adj) < 0.05). Red and blue dots represent upregulated and downregulated genes, respectively, in the MPXV-infected lung organoids. (**C**) Bar plots showing the relative expression levels of lung markers in lung organoids (mock = 1.0). One-way ANOVA followed by the Tukey's post hoc test. Data are shown as means ± SD (*n* = 3).

Next, we examined the effects of MPXV infection on lung organoids. H&E staining revealed no histological changes between the MPXV-infected and uninfected lung organoids (Fig. 2A). To further investigate the impact of MPXV infection on lung organoids, we analyzed host gene expression profiles using RNA-seq. The results showed that infection with MPXV clade Ia, IIa, and IIb significantly altered the expression levels of 656, 249, and 139 genes, respectively (Fig. 2B–Table S2). In our previous analysis using colon organoids [11], MPXV clade Ia induced more pronounced changes in host gene expression profiles compared to other MPXV clades. Taken together, these findings suggest that MPXV clade Ia may have a greater capacity to alter host cell gene expression than other MPXV clades. We examined changes in the expression levels of lung markers and found no significant differences in the expression levels of the goblet cell marker *Mucin 5B* (*MUC5B*), the club cell marker *Secretoglobin Family 1A Member 1* (*SCGB1A1*), the ciliated cell marker *Forkhead Box J1* (*FOXJ1*), the basal cell marker *Tumor Protein P63* (*TP63*), and the neuroendocrine cell marker *Calcitonin Gene-Related Peptide Alpha* (*CALCA*) (Fig. 2C–S1A). These results suggest that MPXV infection did not alter the expression levels of lung markers in lung organoids.

### 3.3 MPXV infection hardly induced the innate immune and inflammatory response in lung organoids

**Fig. 3:**
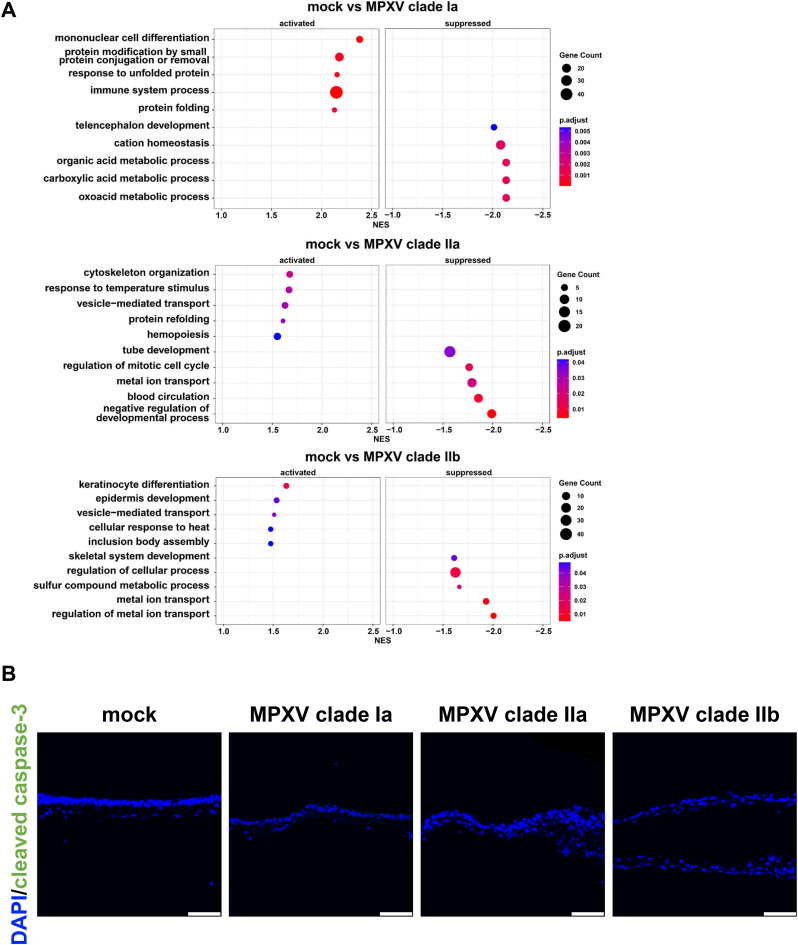
Analysis of differentially expressed genes in MPXV-infected lung organoids Human iPS cell-derived lung organoids were infected with MPXV (0.05 PFU/cell) and cultured for 72 hpi. RNA-seq analysis was performed in uninfected and MPXV-infected lung organoids. (**A**) A dot plot showing the five terms with the highest normalized enrichment scores (NES) and the five terms with the lowest NES, based on the results of gene set enrichment analysis (GSEA). **(B)** Immunofluorescence analysis of cleaved Caspase-3 (green) in uninfected and MPXV-infected lung organoids. Nuclei were counterstained with DAPI (blue). Scale bars 50 μm.

To investigate whether MPXV infection induces innate immune and inflammatory responses in lung organoids, we conducted RNA-seq analysis and inflammatory cytokine quantification. To investigate the biological processes associated with the differentially expressed genes (DEGs), we performed gene set enrichment analysis (GSEA) using the DEG lists for each condition. Genes associated with “mononuclear cell differentiation” and “immune system process” were significantly enriched among the upregulated genes in the MPXV clade Ia-infected lung organoids (Fig. 3A). However, terms related to innate immune and inflammatory responses were not enriched in lung organoids infected with any of the MPXV clades. Consistently, we found that the expression levels of toll-like receptors (TLRs) and interferon stimulated genes (ISGs) (Fig. S2A) were not changed. In addition, immunostaining analysis revealed that the cleaved Caspase-3-positive cells were hardly observed both in uninfected and MPXV-infected lung organoids, suggesting that MPXV infection did not induce apoptosis in lung organoids (Fig. 3B).

**Fig. 4:**
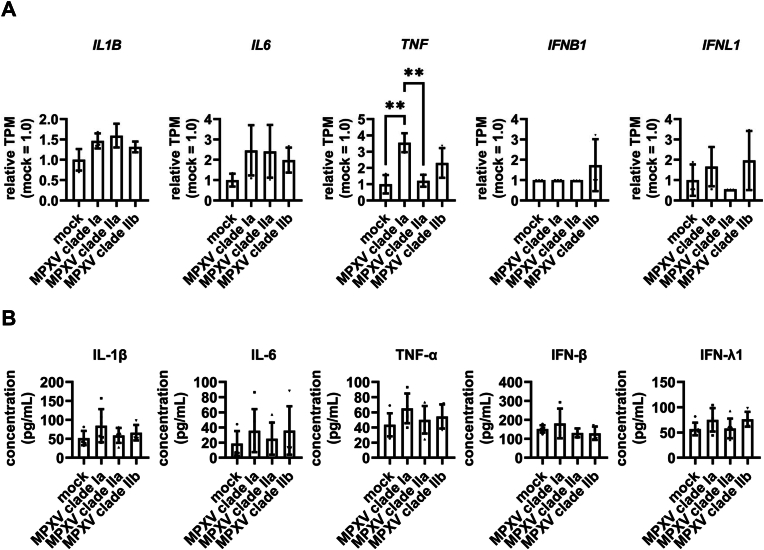
MPXV infection induces little immune response in lung organoids (**A**) Bar plots showing the relative expression levels of innate immune response marker genes in lung organoids (mock = 1.0). One-way ANOVA followed by the Tukey's post-hoc test (∗∗*p* < 0.01). Data are shown as means ± SD (*n* = 3). (**B**) Cytokine productions in the cell culture supernatant of uninfected and MPXV-infected lung organoids were measured by Legend-plex assay. One-way ANOVA followed by the Tukey's post hoc test. Cytokine concentrations are indicated as means ± SD (*n* = 6).

When we examined the expression levels of inflammatory cytokines and interferons, we found that the expression levels of *interleukin-1 beta* (*IL-1β*), *interleukin-6* (*IL-6*), *tumor necrosis factor* (*TNF-α*), *interferon beta* (*IFN-β*), and *interferon lambda-1* (*IFN-λ1*) were not altered by MPXV infection (Fig. 4A, S2B). Consistent with this, we measured the concentrations of interferons and proinflammatory cytokines in the culture supernatants of MPXV-infected lung organoids and found that MPXV infection did not affect the production of either cytokine (Fig. 4B). These results suggest that while MPXV infection can replicate efficiently in lung organoids, it hardly induces innate immune and inflammatory responses.

## 4 Discussion

We demonstrated that all three MPXV strains replicate efficiently in lung organoids. In contrast, MPXV infection hardly induced innate immune and inflammatory responses in lung organoids. This is thought to be due to the presence of an inhibitor of the host immune response encoded by MPXV. Poxviruses encode various proteins that inhibit immune responses [18], such as OPG188 (B2R, poxin), which has been shown to inhibit DNA sensing by cyclic GMP-AMP synthase (cGAS) [19]. In addition, OPG106 (H1L) is known to inhibit interferon signaling and the expression of interferon-stimulated genes (ISGs) by dephosphorylating signal transducer and activator of transcription 1 (STAT1) [20]. Furthermore, it has been reported that the MPXV OPG002 gene encodes a secreted viral TNF receptor (vTNFR), known as cytokine response modifier B (CrmB), which inhibits the activity of the inflammatory cytokine TNF [21]. Functional analysis of these inhibitors of the host immune response may provide insight into why a weak immune response was observed in lung organoids following MPXV infection.

We found that infection with either MPXV clade did not significantly alter the gene expressions of lung markers and innate immune response markers in lung organoids. Therefore, our model appears to recapitulate the pathology observed in mpox patients who did not develop respiratory symptoms. In the future, the validity of our model will be further confirmed by comparing the results obtained from lung organoids with those derived from lung biopsies of mpox patients. However, since lung biopsy sampling from MPXV patients is invasive and poses risks to operators, conducting experiments to analyze differences between MPXV clades using human samples will be challenging.

In this study, we used human iPS cell-derived lung organoids to investigate the effects of MPXV infection on respiratory tissue. However, because these organoids retain fetal-like characteristics, the results should be interpreted with this limitation. Additionally, the organoids lack a vascular network and do not replicate the mechanical stimuli present in the respiratory tissue, resulting in some differences from *in vivo* lung tissue. Therefore, it is essential to recognize and account for these limitations when using organoid models in MPXV research.

As reported by Satapathy et al. [22], the manifestation of respiratory symptoms varies among mpox patients. Therefore, future studies examining inter-individual differences in mpox pathophysiology will be essential. Factors influencing inter-individual differences in pathophysiology include environmental factors such as nutritional status, sanitary conditions, human immunodeficiency virus (HIV) infection, and access to medical care. Additionally, the patient's genetic background, including gender, race, and blood type, is also considered an important factor. Because iPS cells can be derived from almost any individual and reflect their genetic background [23], we believe that a panel of lung organoids derived from iPS cells of diverse individuals could serve as a valuable resource for recapitulating inter-individual differences in host responses to MPXV infection.

## Funding sources

This work was supported by the iPS Cell Research Fund, the Japan Agency for Medical Research and Development (AMED) (JP21gm1610005, JP23fk0108583, JP23jf0126002, JP24fk0108907), and JSPS Core-to-Core Program (A. Advanced Research Networks, JPJSCCA20240006).

## CRediT authorship contribution statement

**Yoshitaka Nakata:** Data curation, Formal analysis, Investigation, Visualization, Writing – original draft. **Keiya Uriu:** Data curation, Investigation. **Rina Hashimoto:** Data curation, Writing – review & editing. **Takuya Yamamoto:** Data curation. **Akatsuki Saito:** Data curation, Funding acquisition. **Kei Sato:** Funding acquisition, Project administration, Writing – review & editing. **Kazuo Takayama:** Conceptualization, Funding acquisition, Project administration, Writing – review & editing.

## Declaration of competing interest

The authors declare the following financial interests/personal relationships which may be considered as potential competing interests:K.S. has consulting fees from Moderna Japan Co., Ltd. and Takeda Pharmaceutical Co. Ltd. and honoraria for lectures from Gilead Sciences, Inc., Moderna Japan Co., Ltd., and Shionogi & Co., Ltd. The other authors declare no competing interests.

## References

[1] Beer E.M., Rao V.B. (2019). A systematic review of the epidemiology of human monkeypox outbreaks and implications for outbreak strategy. PLoS Neglected Trop. Dis..

[2] Bunge E.M., Hoet B., Chen L., Lienert F., Weidenthaler H., Baer L.R., Steffen R. (2022). The changing epidemiology of human monkeypox-A potential threat? A systematic review. PLoS Neglected Trop. Dis..

[3] Nuzzo J.B., Borio L.L., Gostin L.O. (2022). The WHO declaration of monkeypox as a global public health emergency. JAMA.

[4] Pourriyahi H., Aryanian Z., Afshar Z.M., Goodarzi A. (2023). A systematic review and clinical atlas on mucocutaneous presentations of the current monkeypox outbreak: with a comprehensive approach to all dermatologic and nondermatologic aspects of the new and previous monkeypox outbreaks. J. Med. Virol..

[5] Li P., Li J., Ayada I., Avan A., Zheng Q., Peppelenbosch M.P., de Vries A.C., Pan Q. (2023). Clinical features, antiviral treatment, and patient outcomes: a systematic review and comparative analysis of the previous and the 2022 mpox outbreaks. J. Infect. Dis..

[6] Peiró-Mestres A., Fuertes I., Camprubí-Ferrer D., Marcos M., Vilella A., Navarro M., Rodriguez-Elena L., Riera J., Català A., Martínez M.J., Blanco J.L. (2022). Frequent detection of monkeypox virus DNA in saliva, semen, and other clinical samples from 12 patients, Barcelona, Spain, may to June 2022. Euro Surveill..

[7] Gaspari V., Rossini G., Robuffo S., Rapparini L., Scagliarini A., Mistral De Pascali A., Piraccini B.M., Lazzarotto T. (2023). Monkeypox outbreak 2022: clinical and virological features of 30 patients at the sexually transmitted diseases Centre of Sant' Orsola Hospital, Bologna, Northeastern Italy. J. Clin. Microbiol..

[8] Cordeiro R., Pelerito A., de Carvalho I.L., Lopo S., Neves R., Rocha R., Palminha P., Verdasca N., Palhinhas C., Borrego M.J., Manita C., Ferreira I., Bettencourt C., Vieira P., Silva S., Água-Doce I., Roque C., Cordeiro D., Brondani G., Santos J.A., Martins S., Rodrigues I., Ribeiro C., Núncio M.S., Gomes J.P., Batista F.D.C. (2024). An overview of monkeypox virus detection in different clinical samples and analysis of temporal viral load dynamics. J. Med. Virol..

[9] Edman-Wallér J., Jonsson O., Backlund G., Muradrasoli S., Sondén K. (2023). Results of PCR analysis of mpox clinical samples, Sweden, 2022. Emerg. Infect. Dis..

[10] Rosa R.B., Ferreira de Castro E., Vieira da Silva M., Paiva Ferreira D.C., Jardim A.C.G., Santos I.A., Marinho M.D.S., Ferreira França F.B., Pena L.J. (2023). In vitro and in vivo models for monkeypox. iScience.

[11] Watanabe Y., Kimura I., Hashimoto R., Sakamoto A., Yasuhara N., Yamamoto T., Sato K., Takayama K. (2023). Virological characterization of the 2022 outbreak-causing monkeypox virus using human keratinocytes and colon organoids. J. Med. Virol..

[12] Li P., Pachis S.T., Xu G., Schraauwen R., Incitti R., de Vries A.C., Bruno M.J., Peppelenbosch M.P., Alam I., Raymond K., Pan Q. (2023). Mpox virus infection and drug treatment modelled in human skin organoids. Nat. Microbiol..

[13] Chailangkarn T., Teeravechyan S., Attasombat K., Thaweerattanasinp T., Sunchatawirul K., Suwanwattana P., Pongpirul K., Jongkaewwattana A. (2022). Monkeypox virus productively infects human induced pluripotent stem cell-derived astrocytes and neural progenitor cells. J. Infect..

[14] Hashimoto R., Tamura T., Watanabe Y., Sakamoto A., Yasuhara N., Ito H., Nakano M., Fuse H., Ohta A., Noda T., Matsumura Y., Nagao M., Yamamoto T., Fukuhara T., Takayama K. (2023). Evaluation of broad anti-coronavirus activity of autophagy-related compounds using human airway organoids. Mol. Pharm..

[15] Hashimoto R., Watanabe Y., Keshta A., Sugiyama M., Kitai Y., Hirabayashi A., Yasuhara N., Morimoto S., Sakamoto A., Matsumura Y., Nishimura H., Noda T., Yamamoto T., Nagao M., Takeda M., Takayama K. (2025). Human iPS cell-derived respiratory organoids as a model for respiratory syncytial virus infection. Life Sci. Alliance.

[16] Nakagawa M., Taniguchi Y., Senda S., Takizawa N., Ichisaka T., Asano K., Morizane A., Doi D., Takahashi J., Nishizawa M., Yoshida Y., Toyoda T., Osafune K., Sekiguchi K., Yamanaka S. (2014). A novel efficient feeder-free culture system for the derivation of human induced pluripotent stem cells. Sci. Rep..

[17] Gu Z., Eils R., Schlesner M. (2016). Complex heatmaps reveal patterns and correlations in multidimensional genomic data. Bioinformatics.

[18] Hernaez B., Alcamí A. (2024). Poxvirus immune evasion. Annu. Rev. Immunol..

[19] Eaglesham J.B., Pan Y., Kupper T.S., Kranzusch P.J. (2019). Viral and metazoan poxins are cGAMP-specific nucleases that restrict cGAS-STING signalling. Nature.

[20] Mann B.A., Huang J.H., Li P., Chang H.C., Slee R.B., O'Sullivan A., Anita M., Yeh N., Klemsz M.J., Brutkiewicz R.R., Blum J.S., Kaplan M.H. (2008). Vaccinia virus blocks Stat1-dependent and Stat1-independent gene expression induced by type I and type II interferons. J. Interferon Cytokine Res..

[21] Gileva I.P., Nepomnyashchikh T.S., Antonets D.V., Lebedev L.R., Kochneva G.V., Grazhdantseva A.V., Shchelkunov S.N. (2006). Properties of the recombinant TNF-binding proteins from variola, monkeypox, and cowpox viruses are different. Biochim. Biophys. Acta.

[22] Satapathy P., Khatib M.N., Gaidhane S., Zahiruddin Q.S., Alrasheed H.A., Al-Subaie M.F., Al Kaabi N.A., Garout M., Alfaresi M., Sulaiman T., Rabaan A.A., Krsak M., Henao-Martinez A.F., Franco-Paredes C., Serhan H.A., Sah R. (2024). Multi-organ clinical manifestations of Mpox: an umbrella review of systematic reviews. BMC Infect. Dis..

[23] Takahashi K., Tanabe K., Ohnuki M., Narita M., Ichisaka T., Tomoda K., Yamanaka S. (2007). Induction of pluripotent stem cells from adult human fibroblasts by defined factors. Cell.

