# Supplementary material for: Mpox virus replicates in lung organoids without significantly affecting their cellular function
Source: Biochem Biophys Rep. 2025 Nov 7;44:102326. doi: 10.1016/j.bbrep.2025.102326 (PMC12803792; doi:10.1016/j.bbrep.2025.102326)
Supplement: Multimedia component 1 [file mmc1.docx]

**Table S1. List of *p* values of Figures 1B and 1C.**

**Figure 1B**

| **Tukey's multiple comparison test** | **Adjusted *P* value** |
| --- | --- |
| MPXV clade Ia vs MPXV clade IIa | 0.1326 |
| MPXV clade Ia vs MPXV clade IIb | 0.8241 |
| MPXV clade IIa vs MPXV clade IIb | 0.4110 |

**Figure 1C**

| **Tukey's multiple comparison test** | **Adjusted *P* value** |
| --- | --- |
| MPXV clade Ia vs MPXV clade IIa | 0.9948 |
| MPXV clade Ia vs MPXV clade IIb | 0.5712 |
| MPXV clade IIa vs MPXV clade IIb | 0.5189 |

**Table S2. List of differentially expressed genes, related to Figure 2B.**

**mock vs MPXV clade Ia, upregulated**

| Gene | log2FoldChange | *P*adj |
| --- | --- | --- |
| ABCB1 | 1.692002051 | 0.000152458 |
| ABL2 | 1.541218816 | 1.00499E-29 |
| ABTB2 | 1.280820593 | 2.26602E-13 |
| ACTN2 | 1.613604594 | 5.59466E-05 |
| ADAM8 | 1.057463967 | 0.00532255 |
| ADPRHL1 | 3.118059588 | 2.16295E-43 |
| ADRA2B | 1.646263216 | 0.023869214 |
| ALKAL2 | 2.383175087 | 0.027940751 |
| ALMS1-IT1 | 1.281994264 | 0.027428381 |
| ALOXE3 | 2.666606252 | 9.00039E-16 |
| AMHR2 | 1.324066337 | 0.001256805 |
| AQP3 | 1.099279551 | 7.18335E-08 |
| ARC | 6.647391899 | 1.67906E-82 |
| ARHGEF38 | 1.195607471 | 0.023324152 |
| ARL14 | 3.670690116 | 2.89537E-07 |
| ASNS | 1.834787983 | 7.75102E-08 |
| ATAD3C | 1.320879846 | 0.020134197 |
| ATF3 | 1.808653617 | 3.7345E-18 |
| BAG3 | 1.399852696 | 3.40511E-40 |
| BBOX1 | 1.872498843 | 0.022725242 |
| BEAN1 | 1.057707928 | 0.038764238 |
| BEND4 | 1.830398582 | 2.74187E-18 |
| BIRC3 | 1.32296264 | 9.67732E-12 |
| BNC1 | 2.110430708 | 0.000210968 |
| C11orf96 | 2.424886028 | 4.32555E-38 |
| C5AR2 | 1.449703452 | 0.003445438 |
| C6orf141 | 1.649991699 | 7.18583E-09 |
| CALCB | 1.53553164 | 0.001841093 |
| CAPNS2 | 1.513866737 | 0.039719129 |
| CASS4 | 1.302742142 | 0.045487429 |
| CCDC187 | 1.139314856 | 0.001765682 |
| CCL20 | 1.699312364 | 2.64634E-06 |
| CCL22 | 1.622178356 | 0.035061395 |
| CCM2L | 1.218439257 | 0.008629612 |
| CCN1 | 1.489983209 | 1.68366E-10 |
| CCN2 | 1.164926063 | 6.90659E-09 |
| CD247 | 1.410278542 | 0.004857319 |
| CDHR1 | 1.222857419 | 0.007745904 |
| CFLAR-AS1 | 3.404987051 | 0.012781748 |
| CHORDC1 | 1.713920851 | 5.00713E-79 |
| CHRNG | 2.271389035 | 0.016123831 |
| CLDN11 | 1.72274029 | 0.040160404 |
| CREB5 | 1.551648273 | 9.15926E-30 |
| CREBRF | 1.121548575 | 1.74221E-12 |
| CSF3 | 2.412471541 | 2.7718E-06 |
| CSRNP1 | 1.282028414 | 3.32123E-12 |
| CSTA | 1.416437229 | 0.048690573 |
| CXCL2 | 1.920503908 | 1.45635E-08 |
| CXCL8 | 2.672437112 | 3.89158E-16 |
| CYTOR | 1.361443643 | 1.56389E-06 |
| DCAF4L1 | 1.689865297 | 0.026898846 |
| DCLK1 | 1.891532968 | 4.02918E-17 |
| DDX47 | 1.5764703 | 6.45291E-07 |
| DGKH | 1.120768511 | 2.02023E-05 |
| DKK1 | 1.052666419 | 0.000836043 |
| DMBX1 | 1.308759557 | 0.030147007 |
| DMRTA1 | 1.27077682 | 0.020074275 |
| DNAJA4 | 4.772780396 | 1.8383E-192 |
| DNAJB1 | 3.088378895 | 4.7101E-134 |
| DUSP1 | 1.893204334 | 2.7343E-34 |
| DUSP2 | 3.893734846 | 1.12283E-36 |
| EGOT | 6.701706151 | 0.000181277 |
| EGR1 | 4.214169118 | 9.8384E-129 |
| EGR3 | 3.644603104 | 3.60338E-40 |
| EHF | 1.145478668 | 0.000818646 |
| FAM110C | 1.369910713 | 1.60079E-09 |
| FAM241A | 1.049796548 | 0.013718738 |
| FBXL14 | 1.023126139 | 1.48748E-08 |
| FERMT3 | 1.613278137 | 0.007635143 |
| FGF18 | 1.501532902 | 4.70976E-06 |
| FKBP4 | 1.258086377 | 1.75857E-60 |
| FLNC | 2.224967625 | 3.06008E-30 |
| FMNL1-DT | 1.450366393 | 0.001417064 |
| FOS | 1.763990375 | 2.58506E-21 |
| FOSB | 3.202214804 | 5.161E-83 |
| FOSL1 | 3.216873819 | 4.58059E-28 |
| FOXI3 | 1.44660629 | 0.000207549 |
| FUT1 | 1.202491424 | 0.003601625 |
| FUT3 | 1.000636974 | 4.11897E-06 |
| FUT5 | 1.339306016 | 0.005281251 |
| FZD8 | 1.748754338 | 1.57927E-12 |
| GADD45B | 1.224025308 | 3.88457E-67 |
| GALNT9 | 1.343375934 | 0.015854412 |
| GAN | 1.054448995 | 3.32401E-16 |
| GC | 2.79407608 | 0.005495027 |
| GCGR | 1.514371738 | 0.030147007 |
| GCNA | 2.106739395 | 4.2606E-06 |
| GEM | 1.572075715 | 1.50647E-12 |
| GJA3 | 2.131508681 | 0.014788743 |
| GNRH1 | 1.123348377 | 0.015989891 |
| GPR3 | 1.509026661 | 3.63231E-05 |
| H1-3 | 4.078706766 | 8.58061E-07 |
| H1-4 | 4.706673535 | 1.03481E-23 |
| H1-5 | 5.836959708 | 4.90977E-08 |
| H2AC11 | 2.107442585 | 9.10792E-05 |
| H2AC12 | 3.54024261 | 3.20047E-05 |
| H2AC20 | 2.716075473 | 7.13015E-05 |
| H2AC21 | 6.102059668 | 0.000333279 |
| H2AC4 | 5.653025866 | 0.000497143 |
| H2AC7 | 1.264987515 | 0.023991104 |
| H2AC8 | 1.636123513 | 5.52709E-06 |
| H2BC18 | 1.197033202 | 0.008279695 |
| H2BC7 | 1.813116129 | 0.033823105 |
| H2BC8 | 1.916183679 | 2.7986E-08 |
| H3C1 | 6.970712576 | 2.30504E-06 |
| H3C11 | 4.380514885 | 1.5326E-06 |
| H3C12 | 3.693640557 | 0.009495185 |
| H3C13 | 3.858760423 | 0.000359486 |
| H3C2 | 2.746232337 | 0.000169371 |
| H3C3 | 7.534827442 | 2.8815E-07 |
| H3C4 | 1.468972044 | 0.002364229 |
| H3C8 | 3.688875553 | 8.57581E-09 |
| H4C13 | 5.713459444 | 0.000326763 |
| H4C16 | 1.109740268 | 0.000459746 |
| H4C2 | 7.448309874 | 2.24534E-07 |
| H4C3 | 6.890304524 | 8.16993E-09 |
| H4C4 | 3.339065124 | 3.00672E-07 |
| H4C5 | 3.85422258 | 5.90119E-15 |
| H4C8 | 1.054551516 | 0.030449289 |
| HAS1 | 1.840187603 | 0.026416978 |
| HCG27 | 2.070254978 | 0.001291278 |
| HHEX | 1.639523178 | 0.015134965 |
| HIVEP3 | 1.342989749 | 5.03925E-13 |
| HS3ST6 | 1.838413899 | 0.006353325 |
| HSP90AA1 | 1.5220889 | 2.98542E-62 |
| HSPA1A | 4.47753365 | 1.8383E-192 |
| HSPA1B | 3.051767127 | 1.5211E-128 |
| HSPA4L | 1.731589384 | 3.81612E-45 |
| HSPA6 | 7.674465381 | 3.092E-184 |
| HSPA8 | 1.066815293 | 4.37103E-16 |
| HSPD1 | 1.438613981 | 9.98424E-56 |
| HSPH1 | 2.194877577 | 5.4854E-107 |
| IER3 | 1.092020698 | 2.95364E-07 |
| IER5 | 1.445243409 | 5.92687E-43 |
| IFIT2 | 1.336851745 | 0.031703054 |
| IKZF3 | 1.89917043 | 0.014094028 |
| IL11 | 3.202547284 | 3.80478E-56 |
| IL23A | 1.280051236 | 0.005105342 |
| IL31RA | 2.480874202 | 0.000272634 |
| INO80D | 1.142845807 | 3.25367E-18 |
| IRAK2 | 1.01022045 | 7.40198E-05 |
| IRF5 | 1.73411452 | 0.035615793 |
| JUNB | 1.708853496 | 1.39066E-27 |
| KCNH7 | 1.428421173 | 0.029542815 |
| KCNV1 | 1.816519532 | 0.003864076 |
| KLF2 | 1.509440422 | 7.71699E-05 |
| KLHL11 | 1.096454672 | 6.04096E-09 |
| KLHL15 | 1.120525517 | 7.08322E-20 |
| KRT16P6 | 3.810498213 | 2.07173E-07 |
| LINC00877 | 1.370588307 | 0.00756632 |
| LINC01275 | 2.862426851 | 0.033228741 |
| LINC01363 | 1.222275328 | 0.038411505 |
| LINC01783 | 4.386820038 | 1.29855E-08 |
| LINC02731 | 2.331478778 | 1.47845E-06 |
| LINC03126 | 4.696000822 | 1.93182E-14 |
| LINC-PINT | 1.238459693 | 0.004293542 |
| LOC100128770 | 6.603139888 | 1.68477E-05 |
| LOC100134391 | 5.72917323 | 4.11553E-06 |
| LOC119746555 | 1.082943307 | 0.035495341 |
| LOC124903940 | 1.138152241 | 0.026753243 |
| LOC124906608 | 2.293984074 | 0.036961996 |
| LSMEM1 | 1.058423249 | 1.39343E-05 |
| LUCAT1 | 2.711275872 | 0.00029217 |
| LVRN | 1.171128794 | 0.014301646 |
| MAB21L4 | 1.121359759 | 0.006354741 |
| MAFA | 1.906016646 | 0.000865062 |
| MAP3K14 | 1.076648487 | 4.26027E-09 |
| MATN1 | 1.955403766 | 0.012744032 |
| MMP10 | 1.564218154 | 6.87086E-05 |
| MMP25 | 2.029406971 | 4.38556E-05 |
| MMRN2 | 1.674902768 | 0.012453709 |
| MYEOV | 1.775575472 | 0.046819451 |
| NEURL3 | 1.497449341 | 0.002722112 |
| NEXN-AS1 | 3.08842595 | 0.000618643 |
| NFATC2 | 1.357533407 | 5.59858E-12 |
| NFKBIZ | 1.781007782 | 1.04132E-17 |
| NGF | 1.20485392 | 1.95196E-05 |
| NR0B1 | 1.67901069 | 0.033269401 |
| NR1D1 | 1.373005567 | 1.90572E-06 |
| NR4A1 | 2.344087783 | 1.41646E-49 |
| NR4A3 | 1.846022797 | 1.17592E-29 |
| NRIP3 | 1.264685948 | 1.77774E-08 |
| PCDH11X | 1.051006223 | 0.04731438 |
| PI3 | 3.031514555 | 1.14857E-05 |
| PLA2G4E | 2.142927321 | 0.031628261 |
| PLIN4 | 1.439714503 | 0.001774507 |
| PMAIP1 | 1.69857015 | 3.40174E-11 |
| PNLDC1 | 2.555888545 | 0.002731661 |
| PNPLA5 | 5.486668808 | 0.000402809 |
| PPP1R15A | 1.446755187 | 7.3487E-20 |
| PTGER4 | 1.847467661 | 1.35972E-06 |
| RAET1L | 4.366083866 | 0.002245681 |
| RC3H1 | 1.137020429 | 4.94196E-17 |
| REL | 1.109579016 | 1.81907E-11 |
| RELT | 1.059338133 | 1.18492E-13 |
| RFPL3S | 1.078015854 | 0.022719634 |
| RGS2 | 1.404328115 | 1.13583E-08 |
| RNASE1 | 1.539094908 | 1.13048E-11 |
| RNF169 | 1.255926116 | 4.17629E-29 |
| RRAD | 1.822370319 | 3.19904E-10 |
| RUNX3 | 1.606606231 | 0.010081999 |
| S100A2 | 2.370639054 | 8.9624E-08 |
| SCARNA9 | 1.740867955 | 0.039115733 |
| SERPINB5 | 2.001829856 | 0.012814865 |
| SERPINB9 | 1.418806032 | 9.86363E-09 |
| SFTPB | 3.179006601 | 3.34678E-05 |
| SHE | 2.860295627 | 4.20015E-06 |
| SIX4 | 1.382137641 | 8.21203E-05 |
| SLC22A1 | 3.588724146 | 3.61608E-05 |
| SLC34A2 | 2.60655449 | 4.27046E-08 |
| SLC6A14 | 1.321371845 | 0.001595178 |
| SLPI | 1.342778415 | 0.019883692 |
| SOCS3 | 1.243163006 | 1.54394E-11 |
| SPHK1 | 1.273154642 | 1.00937E-13 |
| SPMIP1 | 1.408380572 | 0.000711819 |
| SPOCK1 | 1.132295478 | 0.009480395 |
| SPRR1A | 1.78435029 | 0.042817873 |
| SPRR2A | 2.635532659 | 0.00136962 |
| SPRR2E | 1.930520546 | 0.024606604 |
| SPRR3 | 1.35024013 | 0.021768738 |
| STON2 | 1.099519544 | 0.000310325 |
| STX11 | 1.434742031 | 6.41294E-05 |
| SUMO4 | 2.373851828 | 0.004522859 |
| SYDE2 | 1.137004029 | 0.000167633 |
| TAC1 | 3.235021296 | 3.38614E-15 |
| TAMALIN | 1.929358497 | 5.18693E-12 |
| TFPI2 | 1.565134403 | 2.61741E-12 |
| THOC6 | 1.030578762 | 3.96618E-19 |
| THSD1 | 1.37105019 | 0.008642026 |
| TMPRSS9 | 1.949948093 | 8.21203E-05 |
| TNF | 1.969398225 | 0.027586493 |
| TNFAIP3 | 1.435498393 | 1.48373E-07 |
| TNFRSF9 | 3.723680993 | 3.37783E-14 |
| TRIB1 | 1.496918646 | 2.99395E-18 |
| TRIML2 | 1.35574234 | 0.005983656 |
| TRPV3 | 1.434343434 | 6.7584E-13 |
| TSPEAR | 1.721083527 | 0.01771934 |
| TSPYL2 | 1.255241539 | 1.22991E-39 |
| ULBP1 | 1.595707549 | 5.78477E-05 |
| ULBP2 | 1.168002885 | 0.002745141 |
| UPK3B | 1.105554686 | 0.042731865 |
| USP2 | 1.038932729 | 3.35202E-06 |
| VGF | 3.395523635 | 6.58841E-42 |
| WDR72 | 1.062797757 | 0.001895689 |
| XIRP1 | 8.356097814 | 2.88033E-08 |
| YOD1 | 1.06858716 | 2.31297E-14 |
| ZBED9-AS1 | 1.655359424 | 0.005986322 |
| ZBTB43 | 1.217671383 | 3.35801E-24 |
| ZC3H12A | 1.065392932 | 5.40565E-18 |
| ZFAND2A | 1.432608022 | 1.05292E-15 |
| ZNF286B | 1.033834192 | 0.007145864 |
| ZNF460 | 1.158277154 | 4.05971E-06 |

**mock vs MPXV clade Ia, downregulated**

| Gene | log2FoldChange | *P*adj |
| --- | --- | --- |
| ABCG2 | -1.329297423 | 0.008279112 |
| ACTA2 | -1.334099173 | 0.026900869 |
| ACTG2 | -1.969274444 | 0.000687363 |
| ADCY10 | -1.19306124 | 0.03949881 |
| ADCYAP1R1 | -1.105887823 | 1.1552E-05 |
| ADD2 | -1.014195409 | 0.000156942 |
| ADRA1B | -1.025030643 | 6.69032E-07 |
| AGFG2 | -1.168403533 | 0.005419968 |
| AGMAT | -1.291159303 | 0.035360594 |
| AKR1C1 | -1.603526631 | 0.007995743 |
| ALB | -1.45136876 | 0.039886859 |
| ANGPTL3 | -2.677706689 | 0.012156874 |
| ANXA8 | -1.650633264 | 4.81416E-08 |
| ANXA8L1 | -1.195376555 | 0.003448638 |
| AOAH | -1.164699476 | 0.00338294 |
| APC2 | -1.176842231 | 7.06842E-05 |
| APLNR | -1.900546611 | 9.36517E-05 |
| APOA2 | -1.577730032 | 0.042515364 |
| APOH | -1.808910671 | 0.010170939 |
| ARG1 | -2.841599662 | 0.002529876 |
| ART4 | -1.547452027 | 1.14477E-05 |
| B4GALNT1 | -1.225474933 | 0.010463206 |
| B4GALNT2 | -2.007643501 | 0.048203784 |
| BHMT | -3.257617946 | 7.42739E-05 |
| BMP8A | -1.181841378 | 0.012780818 |
| BMPER | -1.17387751 | 7.73966E-05 |
| BRCA1 | -1.082344248 | 9.58494E-06 |
| BRINP1 | -1.357946002 | 0.001298298 |
| BRINP3 | -2.742858032 | 1.90636E-12 |
| C2CD4A | -1.424895183 | 0.001375969 |
| C2CD4B | -1.582529908 | 0.030628665 |
| C5orf46 | -1.794259856 | 0.020138663 |
| CAMK2N2 | -1.244542111 | 0.043794699 |
| CAPN8 | -1.658315176 | 0.001866003 |
| CARTPT | -2.334330177 | 0.048203784 |
| CASQ1 | -1.284644765 | 0.030752042 |
| CAVIN3 | -1.14809424 | 4.32695E-10 |
| CCDC177 | -1.296736795 | 0.003273019 |
| CCDC183-AS1 | -1.429863966 | 0.009019872 |
| CCDC188 | -1.430591799 | 0.005684017 |
| CCDC190 | -2.356387544 | 0.000454914 |
| CCL15 | -2.145206151 | 0.040804298 |
| CCN4 | -1.847072028 | 2.08472E-18 |
| CDC45 | -1.418881421 | 0.006134982 |
| CDH22 | -1.183931367 | 0.044907009 |
| CDH23 | -1.41719865 | 0.000129397 |
| CDH4 | -1.033357566 | 0.002453473 |
| CDH8 | -1.100399247 | 0.000245319 |
| CDKN2C | -1.18409859 | 0.001593905 |
| CDSN | -1.872981946 | 0.045487429 |
| CDX1 | -1.606009361 | 0.000121317 |
| CEMIP | -1.143947278 | 1.70405E-06 |
| CEND1 | -1.265306842 | 0.012879791 |
| CHGA | -1.56766632 | 0.041381859 |
| CHI3L1 | -1.571946835 | 0.007523454 |
| CHRNB2 | -1.278024669 | 0.000689026 |
| CILP | -2.221670827 | 0.000696063 |
| CNTN2 | -1.470996985 | 4.53777E-08 |
| COL15A1 | -2.067990827 | 5.33127E-06 |
| COL5A1 | -1.195388143 | 3.53701E-17 |
| COL5A3 | -1.124805357 | 0.003055622 |
| COL6A6 | -2.204069573 | 0.000537057 |
| COMP | -1.296523734 | 0.000516449 |
| CPB2 | -1.919692523 | 0.021974277 |
| CRB1 | -1.119617444 | 0.025398607 |
| CRH | -1.979737503 | 0.049949701 |
| CRISPLD2 | -1.081249708 | 1.06946E-08 |
| CSPG5 | -1.038221186 | 0.00070998 |
| CST3 | -1.000855091 | 0.000152874 |
| CXCR4 | -1.269520671 | 2.90795E-05 |
| CYP26B1 | -1.015427031 | 1.2035E-05 |
| CYP27B1 | -1.126320498 | 0.019132868 |
| CYP2C18 | -2.405020087 | 0.021651307 |
| DBX1 | -1.044637093 | 0.013773954 |
| DISP3 | -1.027334693 | 0.00125327 |
| DLGAP3 | -1.100495828 | 0.022708684 |
| DLX1 | -1.336216763 | 0.036163466 |
| DPPA3 | -1.828349898 | 0.022662988 |
| DRAXIN | -1.696148624 | 8.10128E-08 |
| DUSP9 | -1.062657314 | 0.03057859 |
| E2F1 | -1.268666066 | 0.000236273 |
| E2F2 | -1.42759128 | 0.000424369 |
| EFCC1 | -1.222196671 | 6.59273E-06 |
| EHBP1-AS1 | -1.13803122 | 7.9088E-06 |
| ELAVL4 | -1.023045278 | 0.006175959 |
| ELMOD1 | -1.795246547 | 0.000596855 |
| EMILIN2 | -1.221526765 | 7.60616E-07 |
| EN1 | -1.004275528 | 0.016406932 |
| ENHO | -1.567071788 | 0.013664188 |
| EXO1 | -1.096563492 | 0.001872022 |
| F2RL2 | -1.270900623 | 0.012931506 |
| FABP1 | -1.767915829 | 0.047343918 |
| FAM181A | -1.026922286 | 0.034368492 |
| FAM20A | -1.402937901 | 0.043323346 |
| FBLN5 | -1.098770308 | 9.14502E-13 |
| FBN1 | -1.098079526 | 4.71091E-11 |
| FBP1 | -1.129795889 | 0.029120844 |
| FER1L6 | -1.151434394 | 0.00652361 |
| FGF7 | -1.368753527 | 0.028318196 |
| FGFBP3 | -1.420434272 | 0.036581112 |
| FMO3 | -3.631213317 | 0.012168493 |
| FMO5 | -1.984667343 | 0.031781228 |
| FNDC5 | -1.047932421 | 0.004891368 |
| FOXF1 | -1.608858711 | 0.007939729 |
| GABBR2 | -1.708125426 | 5.20517E-05 |
| GABRP | -1.39167379 | 2.08436E-12 |
| GATA3-AS1 | -1.099220678 | 0.048198484 |
| GBX2 | -2.065325284 | 0.007499891 |
| GCG | -2.590757365 | 0.004694505 |
| GDF6 | -1.145118709 | 2.56064E-07 |
| GGT5 | -1.232813598 | 7.21984E-07 |
| GHRHR | -2.114100462 | 0.022863012 |
| GJA5 | -1.342656654 | 0.004450589 |
| GLIPR1 | -1.287647129 | 8.12009E-05 |
| GNA14 | -1.777164937 | 3.42233E-08 |
| GP2 | -1.482108443 | 0.010457058 |
| GPC1-AS1 | -1.009378374 | 0.006045698 |
| GPR139 | -3.208200408 | 0.035667111 |
| GPR149 | -2.096435353 | 0.031868917 |
| GRIK3 | -1.12505525 | 0.018069776 |
| GRM8 | -1.09287671 | 0.0001031 |
| GSTA1 | -2.326126483 | 0.003588392 |
| GSTA2 | -2.033149676 | 0.001937158 |
| GUCY1A1 | -1.363322392 | 7.74769E-12 |
| HAL | -1.478725744 | 0.023676557 |
| HAND1 | -1.870796289 | 0.001337741 |
| HEPHL1 | -2.864607797 | 0.026884092 |
| HGF | -1.092700654 | 0.046434545 |
| HMCN2 | -1.368337767 | 0.005797415 |
| HMGCS2 | -2.550220436 | 0.010537737 |
| HOXA11 | -1.413253349 | 0.004715816 |
| HOXA11-AS | -2.454333221 | 0.023974107 |
| HOXA13 | -1.727698552 | 0.002131519 |
| HOXA2 | -1.5511072 | 0.020416863 |
| HOXA3 | -1.136982536 | 0.031742997 |
| HOXA4 | -1.873710067 | 0.003098395 |
| HOXD12 | -3.228728627 | 0.006864218 |
| HSD17B6 | -1.371392895 | 0.041368129 |
| HSD3B1 | -1.675365536 | 0.00068149 |
| HSPB6 | -2.430170142 | 7.29051E-11 |
| HTRA3 | -1.362173747 | 0.002211466 |
| IGFBP3 | -1.162946488 | 6.16539E-15 |
| IGFL2-AS1 | -1.78233523 | 0.022238922 |
| IL17RB | -1.081148878 | 0.033566907 |
| IL18R1 | -1.55375619 | 0.000142121 |
| IL22RA1 | -1.273541683 | 0.02891288 |
| IL33 | -1.317157712 | 0.03057859 |
| INS | -1.086327828 | 0.03057859 |
| ISM2 | -1.039475062 | 0.038867083 |
| ITIH3 | -2.125204901 | 0.04718747 |
| JAML | -1.416294614 | 0.028982029 |
| JPH2 | -1.811018476 | 8.1718E-12 |
| KCNE3 | -1.048272111 | 0.000706063 |
| KCNE4 | -1.301627186 | 0.017924635 |
| KCNK12 | -1.00578164 | 0.001952426 |
| KCNMB3 | -1.191452194 | 0.007444673 |
| KCNQ2 | -1.029734943 | 0.001838792 |
| KIAA1549L | -1.297447423 | 4.65193E-05 |
| KLHDC8A | -1.260809284 | 0.001522399 |
| KLHL4 | -1.025563592 | 0.000238718 |
| LGR5 | -1.295248761 | 6.85866E-07 |
| LHX2 | -2.938169623 | 1.70738E-06 |
| LHX9 | -1.744070417 | 0.005361801 |
| LINC00842 | -1.749727474 | 0.000984472 |
| LINC01159 | -1.514391241 | 0.012073236 |
| LINC01224 | -1.131748108 | 0.005879562 |
| LINC01270 | -1.81931074 | 0.022453038 |
| LINC01559 | -1.442182422 | 0.006356319 |
| LINC02735 | -1.405756754 | 0.048796279 |
| LIPF | -2.685179046 | 0.001093509 |
| LIX1 | -1.232525525 | 1.57312E-09 |
| LOC124903770 | -2.237379945 | 0.006930182 |
| LOC154761 | -1.544623871 | 0.030112101 |
| LOC343052 | -2.92728823 | 0.036260125 |
| LRFN5 | -1.210794748 | 1.7512E-07 |
| LRRC3B | -1.108796158 | 0.038932506 |
| LRRC55 | -1.042185319 | 8.30332E-05 |
| MAGEA11 | -1.239002982 | 0.030410017 |
| MAPK4 | -1.22385845 | 0.030686587 |
| MASP1 | -1.144258067 | 0.02804117 |
| MCM10 | -1.579788483 | 5.02067E-05 |
| MFAP4 | -1.111288361 | 0.000650574 |
| MFAP5 | -1.082675438 | 2.32506E-05 |
| MFSD13A | -1.085989379 | 0.009494265 |
| MIR124-1HG | -1.892662859 | 0.001141601 |
| MIR124-2HG | -1.083757525 | 0.000102117 |
| MIR219A2HG | -5.300329662 | 0.002280787 |
| MIR9-2HG | -2.244325702 | 0.001298298 |
| MIR9-3HG | -1.581185717 | 0.006016177 |
| MIRLET7IHG | -1.301598848 | 0.001895689 |
| MMP9 | -1.577124307 | 0.000251145 |
| MTA1-DT | -1.583324661 | 0.007384656 |
| MVP-DT | -1.075329033 | 0.008642026 |
| MYBL2 | -1.285278993 | 0.000298 |
| MYBPC1 | -1.713571975 | 0.041708416 |
| MYLK | -1.670886115 | 4.35858E-13 |
| MYO1A | -1.667200116 | 0.015858923 |
| MYT1L | -1.375524778 | 0.03476618 |
| NCAN | -1.001754081 | 0.022005238 |
| NDST3 | -1.865155946 | 2.02023E-05 |
| NELL1 | -1.002791466 | 0.006612688 |
| NES | -1.015412878 | 1.24657E-06 |
| NEUROD4 | -1.156408456 | 0.012993318 |
| NEUROG2 | -2.647951099 | 7.22619E-05 |
| NHLH1 | -1.676535252 | 1.20277E-06 |
| NID2 | -1.141735523 | 2.4543E-08 |
| NIPAL2 | -1.213763259 | 0.000308315 |
| NKAIN3 | -1.02103066 | 0.001053702 |
| NPAS3 | -1.24164408 | 2.64634E-06 |
| NPY | -1.468698936 | 0.002051317 |
| NQO1 | -1.031010445 | 0.009418418 |
| NR1I2 | -1.370931706 | 0.015858923 |
| NR2E1 | -2.598234026 | 1.34306E-11 |
| NTRK2 | -1.232560962 | 0.042856762 |
| NTS | -1.06564394 | 0.030584792 |
| NTSR1 | -1.255851993 | 0.038860778 |
| OLFM4 | -1.496388494 | 0.022877183 |
| OLFML3 | -1.076449925 | 5.65524E-06 |
| OLIG3 | -1.769106219 | 6.49422E-08 |
| ORM1 | -1.376513961 | 0.008642026 |
| OSR1 | -1.648408039 | 1.2776E-12 |
| P2RX3 | -1.012907635 | 0.005714737 |
| P2RY4 | -1.54241745 | 0.004647911 |
| P2RY6 | -1.390621975 | 2.27377E-09 |
| PANTR1 | -1.217486603 | 3.89589E-05 |
| PAPPA2 | -1.338627276 | 1.05231E-09 |
| PCDH20 | -1.082937086 | 0.002620835 |
| PCP4 | -1.968628819 | 0.018240713 |
| PCSK1 | -1.231080242 | 0.039542375 |
| PDE1C | -1.109049053 | 0.03056814 |
| PDZK1 | -1.079743155 | 0.025609312 |
| PITX1 | -1.558551592 | 0.008033305 |
| PLAT | -1.050757677 | 9.85045E-12 |
| PLSCR5 | -1.248691614 | 0.007662269 |
| PLXNA4 | -1.214042312 | 0.002657251 |
| POU3F2 | -1.572303865 | 1.37474E-05 |
| POU3F4 | -1.012539926 | 0.015272462 |
| PRDM8 | -1.009595109 | 0.037000542 |
| PRND | -2.245780599 | 0.000900981 |
| PROK1 | -2.961380476 | 0.000632401 |
| PRR15 | -1.3339484 | 6.9533E-06 |
| PRR16 | -1.274341964 | 0.016123831 |
| PRRT4 | -1.196095667 | 0.02810804 |
| PRSS56 | -2.185215524 | 2.4993E-06 |
| PTGES | -1.151041402 | 2.35731E-10 |
| PTGFR | -1.270128162 | 0.012353675 |
| RBFOX1 | -2.753108279 | 0.003859039 |
| RBP3 | -2.010061328 | 0.001083635 |
| REG4 | -2.358346351 | 0.015509521 |
| RERG | -1.111920122 | 0.000430091 |
| REXO5 | -1.347212574 | 0.01661715 |
| RGN | -1.5330279 | 0.043561964 |
| RGS8 | -1.942099042 | 0.004023919 |
| RHBDL3 | -1.312134527 | 0.000143157 |
| RRM2 | -1.349926062 | 2.51815E-11 |
| S100A3 | -1.409834901 | 0.00931217 |
| S100A4 | -1.074174296 | 5.774E-05 |
| SCGB2A1 | -2.734800668 | 0.048137433 |
| SCML4 | -2.533243075 | 0.00894801 |
| SCN7A | -1.391682595 | 0.009364864 |
| SCRG1 | -2.377522619 | 0.006689005 |
| SDR16C5 | -1.724437678 | 0.003580521 |
| SEMA5B | -1.781057738 | 5.64334E-09 |
| SERPINA7 | -2.443359752 | 0.014514026 |
| SFRP2 | -1.042549731 | 1.81273E-18 |
| SFXN2 | -1.008875584 | 0.000120708 |
| SHD | -1.21426141 | 0.015981194 |
| SHOX2 | -1.421578999 | 0.014856239 |
| SIDT1 | -1.155619394 | 0.045131943 |
| SLC10A1 | -2.587693647 | 0.042049505 |
| SLC17A4 | -1.188411665 | 0.049757304 |
| SLC18A1 | -2.174131485 | 0.001121476 |
| SLC1A2 | -1.299902808 | 2.97072E-05 |
| SLC29A3 | -1.110137884 | 0.000528341 |
| SLC30A2 | -1.108350818 | 0.010504649 |
| SLC4A10 | -1.626355107 | 0.002280877 |
| SLC5A9 | -1.67875577 | 0.018688903 |
| SLC6A11 | -2.163586585 | 0.007269954 |
| SLITRK6 | -1.150268786 | 8.54604E-13 |
| SLN | -2.252139825 | 0.000577203 |
| SMCO3 | -1.833074096 | 8.45808E-05 |
| SMLR1 | -1.828536857 | 0.032570244 |
| SNX10-AS1 | -1.051965271 | 0.028318196 |
| SOWAHA | -1.171479019 | 0.048404588 |
| SOX1 | -1.082385902 | 0.000478067 |
| SP9 | -2.70197952 | 1.30369E-07 |
| SPC24 | -1.174730213 | 8.82833E-05 |
| SPX | -1.215437345 | 0.017715112 |
| ST18 | -1.059364132 | 0.000416812 |
| STBD1 | -1.456005097 | 0.045956964 |
| STEAP1 | -1.578517023 | 0.012657107 |
| SYNDIG1 | -1.036543783 | 2.77211E-06 |
| SYT2 | -1.092732776 | 0.000402809 |
| SYTL2 | -1.115170848 | 0.001593905 |
| TAFA4 | -1.366492158 | 0.003098395 |
| TAT | -2.158117729 | 0.029542815 |
| TBX2 | -1.362426208 | 0.004445898 |
| TCF19 | -1.739000342 | 0.000392009 |
| TCF7L2 | -1.027739292 | 0.000109398 |
| TDO2 | -1.989295556 | 0.004803818 |
| TERT | -1.979453244 | 0.016802369 |
| TGFBI | -1.368199236 | 3.30538E-12 |
| TGM2 | -1.360334598 | 1.87945E-38 |
| TGM4 | -2.222576056 | 0.015579137 |
| THBD | -1.312804396 | 1.21526E-07 |
| TM4SF18-AS1 | -3.182848095 | 0.008012978 |
| TMEM132B | -1.983146474 | 3.79995E-05 |
| TMEM179 | -1.922680517 | 0.002245681 |
| TNFRSF11B | -1.136464892 | 0.020888077 |
| TPH1 | -1.741222825 | 0.025645654 |
| UBASH3B | -1.054625765 | 4.60628E-13 |
| UGT2B11 | -1.504781101 | 0.041301009 |
| VSTM2A | -1.922800508 | 0.001985459 |
| VTCN1 | -1.427734096 | 1.06875E-11 |
| VXN | -1.170729788 | 0.017877551 |
| WDR76 | -1.210097788 | 0.000992434 |
| WNK4 | -1.107562334 | 0.005986322 |
| WNT5A | -1.134996109 | 1.62304E-09 |
| WNT8B | -1.018400856 | 0.000155873 |
| ZBED2 | -1.780178981 | 5.59466E-05 |
| ZG16 | -2.036607317 | 0.031809336 |
| ZNF488 | -1.20840981 | 1.81907E-11 |

**mock vs MPXV clade IIa, upregulated**

| Gene | log2FoldChange | *P*adj |
| --- | --- | --- |
| ABL2 | 1.258795335 | 1.90104E-19 |
| ABTB2 | 1.119341387 | 6.14328E-10 |
| ADPRHL1 | 3.478957931 | 1.96831E-54 |
| ALOXE3 | 2.640980886 | 2.65746E-15 |
| ARC | 5.889360071 | 2.96185E-64 |
| ARL14 | 3.115142812 | 5.82009E-05 |
| ATF3 | 1.765963128 | 3.62177E-17 |
| BAG3 | 1.574265906 | 4.06859E-51 |
| BCO1 | 1.340881106 | 0.035937904 |
| BEND4 | 1.721571117 | 5.85266E-16 |
| BNC1 | 1.421600851 | 0.04956749 |
| C11orf96 | 2.228006314 | 8.47654E-32 |
| C6orf141 | 1.119278954 | 0.00079627 |
| CCN1 | 1.335477648 | 3.81237E-08 |
| CCN2 | 1.110871635 | 8.65914E-08 |
| CFLAR-AS1 | 3.205032167 | 0.043900448 |
| CHORDC1 | 1.478367496 | 4.85228E-58 |
| CREB5 | 1.623219786 | 9.66112E-33 |
| CSRNP1 | 1.030104227 | 1.33158E-07 |
| CSTA | 1.785116239 | 0.016898404 |
| CXCL2 | 1.116998106 | 0.007179596 |
| CXCL8 | 1.270098179 | 0.001688788 |
| CYTOR | 1.381128233 | 1.88659E-06 |
| DCLK1 | 1.460415442 | 7.37722E-10 |
| DDX47 | 1.299936653 | 0.000216077 |
| DKK1 | 1.092521205 | 0.000974212 |
| DNAJA4 | 4.626310425 | 6.8436E-181 |
| DNAJB1 | 2.934838165 | 7.0711E-121 |
| DUSP1 | 1.643759457 | 1.42741E-25 |
| DUSP2 | 3.554519317 | 4.1669E-30 |
| DUSP8 | 1.126458176 | 1.30857E-07 |
| EGOT | 5.621388169 | 0.005934164 |
| EGR1 | 3.700449493 | 6.8171E-99 |
| EGR3 | 3.067838204 | 1.79166E-27 |
| FAM110C | 1.176446408 | 1.00432E-06 |
| FBXL14 | 1.125145906 | 4.1004E-10 |
| FERMT3 | 1.909500852 | 0.00162823 |
| FGF18 | 1.217379733 | 0.000924822 |
| FITM1 | 1.285510554 | 0.045814272 |
| FKBP4 | 1.13590864 | 4.64558E-49 |
| FLNC | 2.36045377 | 4.48591E-34 |
| FMNL1-DT | 1.591630624 | 0.000614577 |
| FOS | 1.308421385 | 2.28502E-11 |
| FOSB | 2.340565012 | 5.5027E-42 |
| FOSL1 | 2.780510298 | 1.62549E-20 |
| FZD8 | 1.292056378 | 1.4613E-06 |
| GALNT9 | 1.500667513 | 0.011054639 |
| GCNA | 1.863566359 | 0.000161498 |
| GEM | 1.254656045 | 1.00512E-07 |
| GPR3 | 1.258080634 | 0.002182278 |
| H1-3 | 4.041089728 | 2.19394E-06 |
| H1-4 | 4.419402928 | 1.20263E-20 |
| H1-5 | 5.507036906 | 7.70007E-07 |
| H2AC11 | 1.828945658 | 0.002277211 |
| H2AC12 | 3.164120846 | 0.000686182 |
| H2AC20 | 2.456297738 | 0.001027193 |
| H2AC21 | 6.379261369 | 0.000296418 |
| H2AC4 | 5.025450256 | 0.005961819 |
| H2AC7 | 1.355345663 | 0.028435458 |
| H2AC8 | 1.58409065 | 2.52406E-05 |
| H2BC8 | 1.449029208 | 0.000197051 |
| H3C1 | 6.331757031 | 6.04644E-05 |
| H3C10 | 1.003147915 | 0.03910323 |
| H3C11 | 4.382264562 | 2.8621E-06 |
| H3C12 | 3.722709009 | 0.018014398 |
| H3C13 | 4.069783259 | 0.000269709 |
| H3C2 | 3.244137646 | 7.01799E-06 |
| H3C3 | 7.077217165 | 4.16402E-06 |
| H3C4 | 1.273077102 | 0.023930759 |
| H3C8 | 3.754329042 | 7.32549E-09 |
| H4C13 | 4.957730046 | 0.006024854 |
| H4C2 | 7.111270412 | 2.00452E-06 |
| H4C3 | 6.507276523 | 1.44309E-07 |
| H4C4 | 3.118578405 | 5.24244E-06 |
| H4C5 | 3.710031468 | 1.41309E-13 |
| HCG27 | 1.758726158 | 0.018977745 |
| HIVEP3 | 1.355938174 | 3.71235E-13 |
| HLA-V | 1.062746752 | 0.011856857 |
| HSP90AA1 | 1.323020297 | 8.20565E-47 |
| HSPA1A | 4.637164592 | 9.4422E-207 |
| HSPA1B | 2.899508633 | 8.9754E-116 |
| HSPA4L | 1.543584336 | 1.50614E-35 |
| HSPA6 | 7.681887851 | 1.9897E-184 |
| HSPB1 | 1.066593638 | 5.25161E-24 |
| HSPD1 | 1.387754993 | 1.13486E-51 |
| HSPH1 | 2.103865643 | 3.66495E-98 |
| IER5 | 1.420475538 | 1.80054E-41 |
| IL11 | 3.017371762 | 1.45292E-49 |
| IL31RA | 3.07492413 | 2.99176E-06 |
| ITGAX | 1.891772242 | 0.007642153 |
| JUNB | 1.167311507 | 2.05199E-12 |
| KLF2 | 1.060929456 | 0.026913004 |
| KRT16P6 | 2.925884361 | 0.000385243 |
| LIF | 1.045734012 | 7.58263E-06 |
| LINC01783 | 4.049513701 | 5.50858E-07 |
| LINC02731 | 2.528084323 | 1.55608E-07 |
| LINC03126 | 4.080082961 | 1.90818E-10 |
| LINC-PINT | 1.076595442 | 0.034370364 |
| LOC100128770 | 6.463164653 | 5.80368E-05 |
| LOC100134391 | 6.577445645 | 9.86236E-08 |
| MAB21L4 | 1.007198136 | 0.034774599 |
| MAFA | 1.419703107 | 0.047218173 |
| MMP10 | 1.196287246 | 0.008827981 |
| MMP25 | 2.288530279 | 3.3854E-06 |
| MMRN2 | 1.690113744 | 0.023484168 |
| NBPF25P | 1.342468897 | 0.000550648 |
| NEXN-AS1 | 2.461847619 | 0.023891128 |
| NFATC2 | 1.103885959 | 1.37885E-07 |
| NFKBIZ | 1.554384332 | 3.34474E-13 |
| NGF | 1.192497863 | 4.88848E-05 |
| NR0B1 | 2.157989234 | 0.006107139 |
| NR4A1 | 1.938653948 | 1.28059E-33 |
| NR4A3 | 1.568976217 | 9.85471E-21 |
| PLA2G4E | 2.241914346 | 0.046493036 |
| PMAIP1 | 1.259880696 | 7.66802E-06 |
| PNLDC1 | 3.007528092 | 0.00040543 |
| PNPLA5 | 5.572769984 | 0.000622784 |
| PPP1R15A | 1.065648017 | 1.85199E-10 |
| PTGER4 | 1.59359543 | 0.000115973 |
| RAET1L | 4.532313219 | 0.002692779 |
| RGS2 | 1.067824111 | 8.30048E-05 |
| RNF169 | 1.019610712 | 9.72406E-19 |
| RRAD | 1.515820369 | 8.83198E-07 |
| S100A2 | 2.076543093 | 1.23399E-05 |
| S100A8 | 1.668689398 | 0.040995028 |
| SERPINB9 | 1.157138069 | 1.5106E-05 |
| SHE | 2.698059757 | 4.15048E-05 |
| SLC22A1 | 3.524181438 | 0.00010926 |
| SLC6A14 | 1.035132285 | 0.041758718 |
| SOCS3 | 1.323223365 | 7.95193E-13 |
| SPHK1 | 1.080906819 | 1.43178E-09 |
| SPMIP1 | 1.649186591 | 7.01179E-05 |
| SPRR1A | 2.397667023 | 0.006928056 |
| SPRR2A | 2.342558335 | 0.012961865 |
| TAC1 | 1.776732732 | 0.000493353 |
| TAMALIN | 1.178516102 | 0.000283654 |
| TFPI2 | 1.600336056 | 1.14023E-12 |
| THOC6 | 1.011096366 | 2.36139E-18 |
| TMPRSS9 | 1.475322737 | 0.013084793 |
| TNFRSF9 | 2.608936532 | 1.88659E-06 |
| TRH | 1.257846762 | 0.00028448 |
| TRIB1 | 1.407919243 | 5.85266E-16 |
| TRIML2 | 1.355558294 | 0.012167991 |
| TRPV3 | 1.093504935 | 3.81798E-07 |
| TSPEAR | 1.708636121 | 0.038589295 |
| TSPYL2 | 1.279214606 | 2.78565E-41 |
| VGF | 3.948609232 | 4.10266E-57 |
| XIRP1 | 7.490928546 | 2.36236E-06 |
| ZBTB43 | 1.069356513 | 2.32526E-18 |
| ZFAND2A | 1.274743062 | 3.83048E-12 |

**mock vs MPXV clade IIa, downregulated**

| Gene | log2FoldChange | *P*adj |
| --- | --- | --- |
| ACTA2 | -1.356735121 | 0.04956749 |
| ACTG2 | -1.80854613 | 0.004412316 |
| ACVRL1 | -1.198646203 | 0.031339565 |
| APLNR | -1.345081487 | 0.018254243 |
| ART4 | -1.133515771 | 0.004991905 |
| BHMT | -2.81589754 | 0.001536604 |
| BRCA1 | -1.055682739 | 3.22814E-05 |
| BRINP1 | -1.067069451 | 0.032699856 |
| BRINP3 | -1.241905757 | 0.003310455 |
| CAPN8 | -1.280298109 | 0.046352299 |
| CCN4 | -1.395845305 | 2.44422E-11 |
| CD248 | -1.17145001 | 0.001729508 |
| CDC45 | -1.771034739 | 0.00089544 |
| CDH20 | -1.674079983 | 0.031002169 |
| CDSN | -2.141376043 | 0.04591339 |
| CDX1 | -1.145882672 | 0.022583607 |
| CILP | -2.032580493 | 0.004412316 |
| CLSPN | -1.134606784 | 9.51398E-05 |
| CMKLR1 | -1.648603054 | 0.013061855 |
| COL15A1 | -1.603894507 | 0.001500655 |
| COL6A6 | -1.866386555 | 0.00833758 |
| COMP | -1.278268953 | 0.00125741 |
| CRISPLD2 | -1.047673609 | 6.81838E-08 |
| DCN | -1.344845198 | 0.000408203 |
| DGCR5 | -1.069710096 | 0.003194659 |
| DTL | -1.203841258 | 0.003898739 |
| E2F1 | -1.209065639 | 0.001037617 |
| E2F2 | -1.675047323 | 5.14088E-05 |
| EMILIN1 | -1.117311728 | 0.014002968 |
| EN1 | -1.255764396 | 0.003829501 |
| FXYD2 | -1.412106019 | 0.042577251 |
| GGT5 | -1.051052256 | 7.04588E-05 |
| GINS2 | -1.051035676 | 0.001042157 |
| GJA5 | -1.344906576 | 0.008393944 |
| GNA14 | -1.475304777 | 8.52334E-06 |
| GUCY1A1 | -1.138771561 | 3.75359E-08 |
| HOXA13 | -1.868188588 | 0.001622069 |
| HSPB6 | -2.361430415 | 3.03082E-10 |
| IL18R1 | -1.199031121 | 0.010993146 |
| ISLR | -1.90626326 | 0.018107097 |
| JPH2 | -1.084043087 | 0.000279956 |
| KCNE4 | -1.330041482 | 0.031361355 |
| KCNMB3 | -1.137935805 | 0.022900804 |
| LINC01963 | -1.05458892 | 0.04816523 |
| LRFN5 | -1.044938659 | 1.44897E-05 |
| MCM10 | -1.161496103 | 0.008390807 |
| MFAP4 | -1.183023728 | 0.000493353 |
| MIRLET7IHG | -1.163866404 | 0.012140665 |
| MYLK | -1.411657261 | 3.04674E-09 |
| NPY | -1.193658384 | 0.032714626 |
| NTNG2 | -1.008441993 | 0.031734569 |
| OSR1 | -1.125164673 | 8.52334E-06 |
| PAPPA2 | -1.303629506 | 5.2413E-09 |
| PCDH20 | -1.034575632 | 0.008777068 |
| PROK1 | -2.742356278 | 0.003257105 |
| PRSS56 | -1.152336508 | 0.028735925 |
| RBP3 | -1.479868321 | 0.043266234 |
| S100A3 | -1.371960292 | 0.022900804 |
| SCGB2A1 | -3.686930136 | 0.019250234 |
| SDR16C5 | -1.42881637 | 0.040303743 |
| SMCO3 | -1.137084731 | 0.037985847 |
| SPC24 | -1.179688441 | 0.000153604 |
| SYNC | -1.428196065 | 0.024026404 |
| TCF19 | -1.426643205 | 0.009793371 |
| TGM4 | -2.135646722 | 0.039803733 |
| TMEM132B | -1.748550422 | 0.00076009 |
| ZBED2 | -1.438873972 | 0.003232638 |
| ZNF575 | -1.616138305 | 0.03628844 |

**mock vs MPXV clade IIb, upregulated**

| Gene | log2FoldChange | *P*adj |
| --- | --- | --- |
| ADPRHL1 | 1.318781825 | 1.20381E-06 |
| ALOXE3 | 1.174950902 | 0.012186698 |
| ARC | 5.923790793 | 8.77413E-65 |
| ASNS | 1.57458992 | 3.54055E-05 |
| C11orf96 | 2.11991007 | 2.15339E-28 |
| CCL20 | 1.007477296 | 0.04869871 |
| CCN1 | 1.028694836 | 0.000173799 |
| CHAC1 | 1.038170932 | 0.016689497 |
| CLCA2 | 1.582638755 | 0.024400123 |
| CSTA | 2.097612221 | 0.004706395 |
| CXCL8 | 1.06099033 | 0.027871595 |
| DCLK1 | 1.025973223 | 0.00017889 |
| DNAJA4 | 2.869983178 | 2.02571E-67 |
| DNAJB1 | 1.732056046 | 3.46686E-41 |
| DUSP1 | 1.162635124 | 6.0031E-12 |
| DUSP2 | 3.455011308 | 4.15048E-28 |
| EGR1 | 2.751942352 | 7.32281E-54 |
| EGR3 | 2.777825257 | 6.98269E-22 |
| FLNC | 1.216782756 | 3.24074E-08 |
| FMNL1-DT | 1.561683153 | 0.001643158 |
| FOSB | 1.34238099 | 6.81395E-12 |
| FOSL1 | 1.928428593 | 5.6241E-09 |
| H1-3 | 3.466349238 | 0.000247569 |
| H1-4 | 3.736372103 | 5.55464E-14 |
| H1-5 | 4.505681759 | 0.000342345 |
| H2AC12 | 2.91611284 | 0.005122733 |
| H2AC20 | 2.306106526 | 0.005394783 |
| H2AC21 | 4.987443247 | 0.023253134 |
| H2AC4 | 5.426146441 | 0.004152785 |
| H2BC18 | 1.237233372 | 0.019459089 |
| H2BC8 | 1.301686726 | 0.002701298 |
| H3C1 | 6.288210047 | 0.000147201 |
| H3C11 | 3.935091876 | 0.00011679 |
| H3C13 | 3.603857135 | 0.0046644 |
| H3C3 | 6.5235706 | 8.46187E-05 |
| H3C8 | 3.188511278 | 6.8677E-06 |
| H4C13 | 4.770865257 | 0.017322151 |
| H4C2 | 6.380992877 | 9.28166E-05 |
| H4C3 | 6.218567425 | 1.71032E-06 |
| H4C4 | 2.520242988 | 0.001515867 |
| H4C5 | 3.284467563 | 4.50916E-10 |
| HSPA1A | 2.864930987 | 1.43173E-75 |
| HSPA1B | 1.775698169 | 2.32362E-42 |
| HSPA6 | 5.651211875 | 2.31678E-98 |
| HSPH1 | 1.105487874 | 5.12914E-26 |
| IL11 | 3.088893047 | 5.60166E-52 |
| IVL | 2.067845746 | 0.000826966 |
| KLF2 | 1.049341845 | 0.045440415 |
| KRT16P6 | 2.751909873 | 0.002372348 |
| LIF | 1.275772063 | 1.67627E-08 |
| LINC01783 | 4.063441553 | 1.04473E-06 |
| LINC03126 | 3.889145689 | 4.92599E-09 |
| LOC100128770 | 4.637255182 | 0.027871595 |
| MMP10 | 1.328185447 | 0.004908942 |
| NR4A1 | 1.425844137 | 2.26415E-17 |
| PMAIP1 | 1.225063705 | 3.34213E-05 |
| RAET1L | 3.936070795 | 0.027871595 |
| S100A2 | 1.946307791 | 0.000124983 |
| S100A8 | 2.158392943 | 0.005627258 |
| SERPINB3 | 1.549272625 | 0.047807541 |
| SERPINB5 | 2.372345996 | 0.008253852 |
| SHE | 2.545525957 | 0.000287627 |
| SLC6A14 | 1.205307001 | 0.018156164 |
| SOCS3 | 1.005830778 | 7.15869E-07 |
| SPRR1A | 3.100248621 | 0.000198919 |
| SPRR2A | 2.536368411 | 0.008617556 |
| SPRR2D | 2.056608482 | 0.003660717 |
| SPRR2E | 2.268984864 | 0.021797626 |
| SPRR3 | 1.735640508 | 0.007311527 |
| TAC1 | 1.671972139 | 0.002701298 |
| TAMALIN | 1.256096093 | 0.000153171 |
| TFPI2 | 1.219596264 | 8.46638E-07 |
| TNFRSF9 | 1.701255439 | 0.018642242 |
| TNNI3 | 1.043314821 | 0.044698702 |
| TRH | 1.202518831 | 0.001379403 |
| VGF | 3.850041466 | 5.71268E-54 |
| XIRP1 | 6.157942192 | 0.00067171 |
| ZNF460 | 1.156478733 | 1.59557E-05 |

**mock vs MPXV clade IIb, downregulated**

| Gene | log2FoldChange | *P*adj |
| --- | --- | --- |
| APLNR | -1.315421029 | 0.036829555 |
| BHMT | -2.647634475 | 0.006788996 |
| BMP8A | -1.229403915 | 0.031712317 |
| BRINP1 | -1.173668539 | 0.024413407 |
| CASQ1 | -1.473280628 | 0.036104314 |
| CCN4 | -1.328514063 | 2.70324E-10 |
| CDC45 | -1.454225731 | 0.015651482 |
| CDH23 | -1.035512366 | 0.032134738 |
| CILP | -1.727784378 | 0.036731858 |
| COL15A1 | -1.481161167 | 0.008457751 |
| COL6A6 | -1.961766491 | 0.00847523 |
| CYSLTR2 | -1.950000619 | 0.009153769 |
| DCN | -1.183469511 | 0.006292227 |
| DTL | -1.247171229 | 0.004702542 |
| E2F1 | -1.049452855 | 0.012126869 |
| E2F2 | -1.491274048 | 0.000721063 |
| EN1 | -1.185975897 | 0.012101559 |
| FXYD2 | -1.569846712 | 0.031039874 |
| GCG | -2.631798874 | 0.014002695 |
| GLIPR1 | -1.199704918 | 0.000754969 |
| GNA14 | -1.206025719 | 0.000754969 |
| GSTA2 | -1.750978142 | 0.032618563 |
| HABP2 | -1.030107908 | 0.028844506 |
| HOXA13 | -1.735771827 | 0.006833686 |
| HSPB6 | -1.848841024 | 1.03155E-06 |
| IL18R1 | -1.261223879 | 0.011276279 |
| JPH2 | -1.049600564 | 0.00098506 |
| KCNK3 | -1.446356792 | 0.045918788 |
| KLHL41 | -1.647097223 | 0.037279698 |
| LUM | -1.1013188 | 5.51329E-06 |
| MCM10 | -1.099789648 | 0.024090703 |
| MYBL2 | -1.121715922 | 0.008350152 |
| MYLK | -1.340300586 | 5.81244E-08 |
| NKX1-2 | -4.102166233 | 0.009284891 |
| OSR1 | -1.039927844 | 0.000123381 |
| PAPPA2 | -1.045751786 | 1.44113E-05 |
| PDE1C | -1.245890019 | 0.04518714 |
| PROK1 | -2.670354721 | 0.007926344 |
| RRM2 | -1.097157414 | 3.65658E-07 |
| SLC18A3 | -1.810730015 | 0.015606397 |
| SPC24 | -1.005569506 | 0.002890927 |
| TDO2 | -1.779116137 | 0.039272584 |
| TGM4 | -2.268937307 | 0.039092299 |
| TMEM132B | -1.490822199 | 0.01204322 |
| TRIM55 | -1.024064049 | 0.007896262 |
| ZBED2 | -1.299807774 | 0.018156164 |

**Table S3. Primers used in this study**

| **Gene** | **Organism** | **Direction** | **Sequence** |
| --- | --- | --- | --- |
| *GAPDH* | human | Fw | GGTGGTCTCCTCTGACTTCAACA |
|  | human | Rv | GTGGTCGTTGAGGGCAATG |
| *MUC5B* | human | Fw | GCCTACGAGGACTTCAACGTC |
|  | human | Rv | CCTTGATGACAACACGGGTGA |
| *SCGB1A1* | human | Fw | TTCAGCGTGTCATCGAAACCC |
|  | human | Rv | ACAGTGAGCTTTGGGCTATTTTT |
| *FOXJ1* | human | Fw | GCCTCCCTACTCGTATGCCA |
|  | human | Rv | GCCGACAGGGTGATCTTGG |
| *TP63* | human | Fw | GTCATTTGATTCGAGTAGAGGGG |
|  | human | Rv | CTGGGGTGGCTCATAAGGT |
| *CALCA* | human | Fw | TCAGCATCTTGGTCCTGTTG |
|  | human | Rv | CTCTCTTGCTCCTGCTCCAG |
| *IL1B* | human | Fw | ATGATGGCTTATTACAGTGGCAA |
|  | human | Rv | GTCGGAGATTCGTAGCTGGA |
| *IL6* | human | Fw | CCTGAACCTTCCAAAGATGGC |
|  | human | Rv | TTCACCAGGCAAGTCTCCTCA |
| *TNF* | human | Fw | CCTCTCTCTAATCAGCCCTCTG |
|  | human | Rv | GAGGACCTGGGAGTAGATGAG |
| *IFNB1* | human | Fw | ATGACCAACAAGTGTCTCCTCC |
|  | human | Rv | GGAATCCAAGCAAGTTGTAGCTC |
| *IFNL1* | human | Fw | CACATTGGCAGGTTCAAATCTCT |
|  | human | Rv | CCAGCGGACTCCTTTTTGG |

**Table S4. Antibodies used in this study**

| **Antigen** | **Catalogue** | **Host** | **Company** |
| --- | --- | --- | --- |
| Donkey anti-Mouse IgG (H+L) Highly Cross-Adsorbed Secondary Antibody, Alexa Fluor Plus 488 | A-21202 | Donkey | Thermo Fisher Scientific |
| Donkey anti-Rabbit IgG (H+L) Highly Cross-Adsorbed Secondary Antibody, Alexa Fluor Plus 488 | A-21206 | Donkey | Thermo Fisher Scientific |
| MPXV A29 Antibody | 40891-M0036 | Mouse | Sino Biological |
| Anti-Cleaved Caspase-3 | 9579 | Rabbit | Cell Signaling Technology |
