# Supplementary material for: Mpox virus replicates in lung organoids without significantly affecting their cellular function
Source: Biochem Biophys Rep. 2025 Nov 7;44:102326. doi: 10.1016/j.bbrep.2025.102326 (PMC12803792; doi:10.1016/j.bbrep.2025.102326)
Supplement: Multimedia component 2 [file mmc2.pdf]

Figure S1

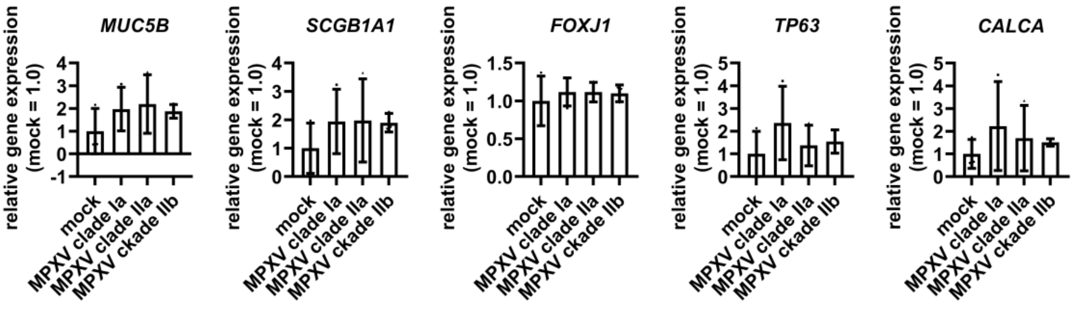

Figure S1. RT-qPCR analysis in MPXV-infected lung organoids

The expression levels of lung markers were measured by RT-qPCR. One-way ANOVA followed by the Tukey post-hoc test. Data are shown as means ± SD ($n = 3$).

Figure S2

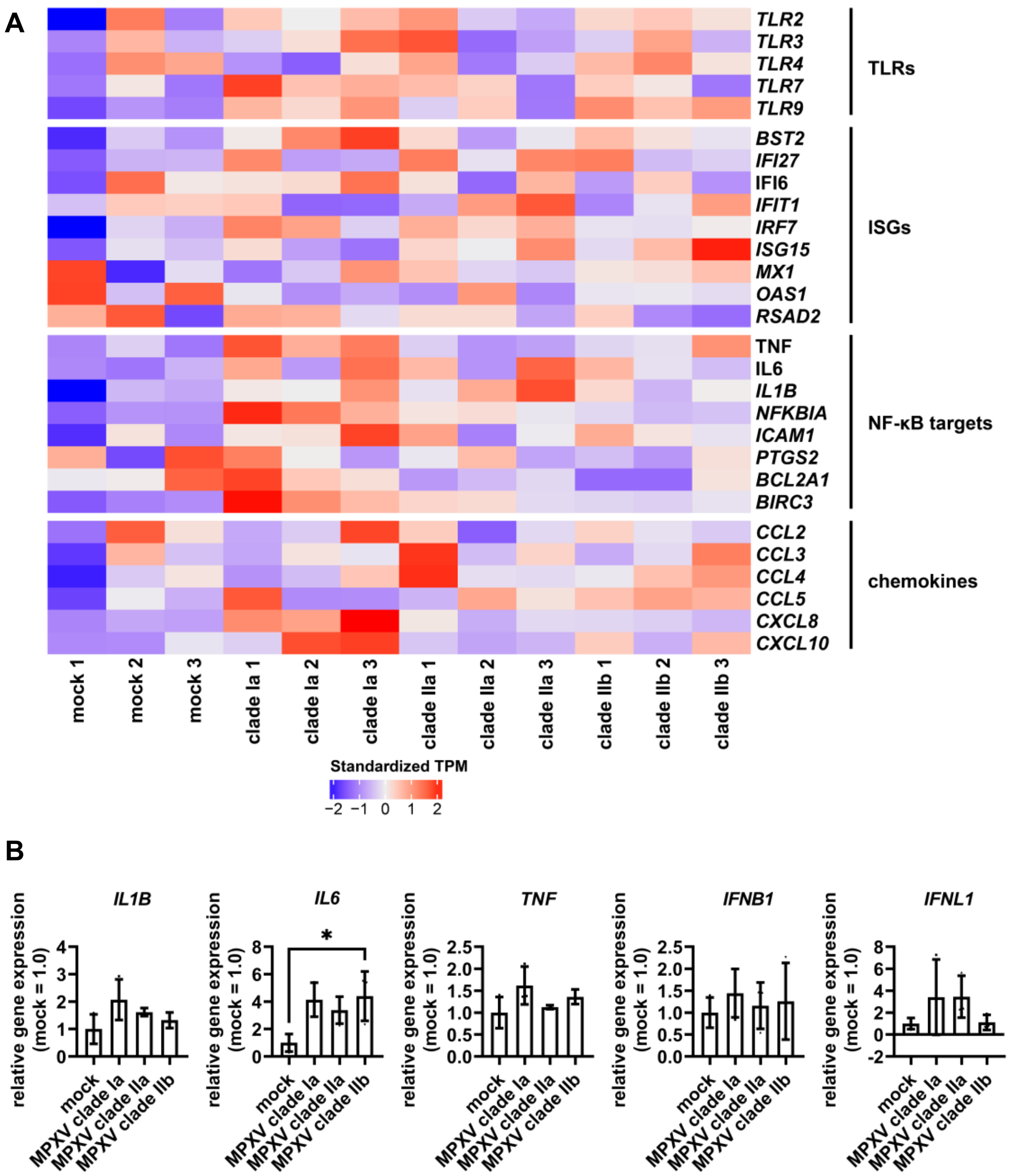

Figure S2. RNA-seq analysis in MPXV-infected lung organoids

(**A**) Heatmap showing the relative expression levels of toll-like receptors (TLRs), interferon stimulated genes (ISGs), nuclear factor-kappa B targets (NF-κB), and chemokines in lung organoids. Standardized transcripts per kilobase million (TPM) values of each gene were visualized. (**B**) The expression levels of innate immune response markers were measured by RT-qPCR. One-way ANOVA followed by the Tukey post-hoc test (*$p < 0.05$). Data are shown as means ± SD ($n = 3$).

Supplemental Materials and Methods

Quantification of viral DNA copy numbers in the cell and culture medium

Viral DNA copy number in cell and culture medium was measured according to our previous report [1].

Immunofluorescence staining

For immunofluorescence staining of human iPS cell-derived lung organoids, the cells were fixed with 4% paraformaldehyde (Cat# 163-20145, FUJIFILM Wako Pure Chemical) for 15 min, harvested, and used to prepare paraffin sections. Paraffin-embedded tissue sections were prepared by the Applied Medical Research Laboratory. Paraffin was removed using xylene and afterward rehydrated with different percentages of ethanol. Antigen retrieval was performed with 0.1%-tTBS (10×) (pH 7.4) (Cat# 12750-81, Nacalai Tesque). For blocking non-specific staining, the slides were incubated in Blocking One Histo (Cat# 06349-64, Nacalai Tesque) for 10 min at room temperature. Primary antibody incubation was performed for 2 hours at room temperature. Stained slides were washed thrice with 1× PBS (Cat# 14249-24, Nacalai Tesque). Alexa Fluor 488-conjugated secondary antibody incubation was performed at room temperature for 45 minutes, and slides were washed afterward three times for 5 min. Sections were washed, mounted with ProLong Glass Antifade Mountant with NucBlue Stain (Cat# P36985, Thermo Fisher Scientific) and DAPI (Cat# 19178-91, Nacalai Tesque), and analyzed using an inverted laser scanning confocal microscopy system (FV3000, Evident). Antibodies used are summarized in **Table S3**.

Hematoxylin & Eosin (H&E) staining

For H&E staining of human iPS cell-derived lung organoids, the cells were fixed with 4% paraformaldehyde for 15 min, harvested, and used to prepare paraffin sections. Paraffin-embedded tissue sectioning and H&E staining were performed by the Applied Medical Research Laboratory.

RNA sequencing

Total RNA was isolated from cells using ISOGENE (Cat# 319-90211, NIPPON GENE). RNA integrity was assessed using a 2100 Bioanalyzer (Agilent Technologies). Library preparation was performed using an Illumina Stranded

mRNA Prep Kit (Cat# 20040532, Illumina) according to the manufacturer’s instructions, including poly(A) selection for mRNA enrichment. Sequencing was performed on an Illumina NextSeq2000 generating an average of 25 million paired-end reads (2x59 bp). The fastq files were generated using bcl2fastq-2.20. Adapter sequences and low-quality bases were trimmed from the raw reads using Cutadapt ver v4.6 [2]. For the transcriptome analysis of human, the trimmed reads were mapped to human reference genome sequences (hg38) using STAR ver 2.7.11a [3] with the GENCODE (release 44, GRCh38.p14) gtf file [4]. For the transcriptome analysis of MPXV, the trimmed reads were mapped to MPXV reference genome sequences of each clade using STAR ver 2.7.10b with these gtf file. BEDtools ver 2.31.1 [5] was used to compute feature coverage from BAM files. The raw counts were calculated using htseq-count ver. 2.0.5 [6] with the GENCODE gtf file. Differential gene expression analysis was performed using DESeq2 v1.42.0 [7] with the raw count data. For visualization and general comparison of gene expression levels, Transcripts Per Kilobase Million (TPM) values were calculated. The differentially expressed genes were visualized as volcano plots using the “EnhancedVolcano” function of the “EnhancedVolcano” package v1.22.0. In **Figures 2C** and **4A**, TPM values of each gene were added by 0.1 and divided by the mean TPM value of uninfected condition to visualize the gene expression as bar plots. In **Figures 1E** and **S2**, TPM values were standardized to visualize the gene expression as heatmap, so that the TPM value of each gene has a mean of 0 and standard deviation of 1. Gene set enrichment analysis (GSEA) was performed by inputting the differentially expressed genes (DEGs) into the “gseGO” function of the “clusterProfiler” package v4.12.0. Since it has been pointed out that up-regulation of histone mRNA is an experimental artifact caused by MPXV infection [8], histone genes were excluded from the DEGs list when performing GSEA. The dot plot showing the result of GSEA was generated by “dotplot” function of the “enrichplot” package v1.24.0. These bioinformatics analyses were performed using R v4.4.0. Raw data generated from this study were submitted under Gene Expression Omnibus (GEO) accession number GSE287262.

RT-qPCR

RT-qPCR was performed according to our previous report [1]. The primer sequences are summarized in **Table S3**.

Measurement of cytokines in cell culture supernatant

The cell culture supernatant was mixed with an equal volume of 1% β-propiolactone (Cat# 168-21011, FUJIFILM Wako Pure Chemical). Cytokine and chemokine concentrations were measured using a LEGENDplex Human Anti-Virus Response Panel (Cat# 740 390, BioLegend). Flow cytometry was performed according to the manufacturer's instructions using MACSQuant Analyzer 10 (Miltenyi Biotec).

Supplemental references

[1] Y. Watanabe, I. Kimura, R. Hashimoto, A. Sakamoto, N. Yasuhara, T. Yamamoto, K. Sato, K. Takayama, Virological characterization of the 2022 outbreak-causing monkeypox virus using human keratinocytes and colon organoids, J Med Virol 95 (2023) e28827. 10.1002/jmv.28827.

[2] M. Martin, Cutadapt removes adapter sequences from high-throughput sequencing reads, EMBnet. journal 17 (2011) 10-12.

[3] A. Dobin, C.A. Davis, F. Schlesinger, J. Drenkow, C. Zaleski, S. Jha, P. Batut, M. Chaisson, T.R. Gingeras, STAR: ultrafast universal RNA-seq aligner, Bioinformatics 29 (2013) 15-21. 10.1093/bioinformatics/bts635.

[4] A. Frankish, M. Diekhans, A.M. Ferreira, R. Johnson, I. Jungreis, J. Loveland, J.M. Mudge, C. Sisu, J. Wright, J. Armstrong, I. Barnes, A. Berry, A. Bignell, S. Carbonell Sala, J. Chrast, F. Cunningham, T. Di Domenico, S. Donaldson, I.T. Fiddes, C. García Girón, J.M. Gonzalez, T. Grego, M. Hardy, T. Hourlier, T. Hunt, O.G. Izuogu, J. Lagarde, F.J. Martin, L. Martínez, S. Mohanan, P. Muir, F.C.P. Navarro, A. Parker, B. Pei, F. Pozo, M. Ruffier, B.M. Schmitt, E. Stapleton, M.M. Suner, I. Sycheva, B. Uszczynska-Ratajczak, J. Xu, A. Yates, D. Zerbino, Y. Zhang, B. Aken, J.S. Choudhary, M. Gerstein, R. Guigó, T.J.P. Hubbard, M. Kellis, B. Paten, A. Reymond, M.L. Tress, P. Flicek, GENCODE reference annotation for the human and mouse genomes, Nucleic Acids Res 47 (2019) D766-d773. 10.1093/nar/gky955.

[5] A.R. Quinlan, I.M. Hall, BEDTools: a flexible suite of utilities for comparing genomic features, Bioinformatics 26 (2010) 841-842. 10.1093/bioinformatics/btq033.

[6] S. Anders, P.T. Pyl, W. Huber, HTSeq--a Python framework to work with high-throughput sequencing data, Bioinformatics 31 (2015) 166-169. 10.1093/bioinformatics/btu638.

[7] M.I. Love, W. Huber, S. Anders, Moderated estimation of fold change and dispersion for RNA-seq data with DESeq2, Genome Biol 15 (2014) 550. 10.1186/s13059-014-0550-8.

[8] D. Bourquain, P.W. Dabrowski, A. Nitsche, Comparison of host cell gene expression in cowpox, monkeypox or vaccinia virus-infected cells reveals virus-specific regulation of immune response genes, Virol J 10 (2013) 61. 10.1186/1743-422x-10-61.
